# The acceptability and feasibility of conducting a randomised controlled trial to test the effectiveness of a walking intervention for older people with persistent musculoskeletal pain in primary care: A mixed methods evaluation of the iPOPP pilot trial

**DOI:** 10.1002/msc.1815

**Published:** 2023-09-09

**Authors:** Emma L. Healey, John McBeth, Elaine Nicholls, Carolyn A. Chew‐Graham, Stephen Dent, Nadine E. Foster, Daniel Herron, Tamar Pincus, Liz Hartshorne, Elaine M. Hay, Clare Jinks

**Affiliations:** ^1^ School of Medicine Keele University Keele Staffordshire UK; ^2^ Arthritis Research UK Centre for Epidemiology The University of Manchester Manchester UK; ^3^ Keele Clinical Trials Unit Keele University Keele Staffordshire UK; ^4^ Midlands Partnership Foundation Trust Stafford Staffordshire UK; ^5^ STARS Education and Research Alliance Surgical Treatment and Rehabilitation Service The University of Queensland and Metro North Health Brisbane Queensland Australia; ^6^ School of Health, Science and Wellbeing Staffordshire University Science Centre Building Stoke‐on‐Trent UK; ^7^ The Faculty for Environment and Life Sciences (FELS) University of Southampton University Road Southampton UK; ^8^ Faculty of Medicine & Health Sciences University of Nottingham Nottingham UK

**Keywords:** data triangulation, musculoskeletal pain, older people, physical activity, primary care, randomised controlled trial, walking

## Abstract

**Introduction:**

Persistent musculoskeletal (MSK) pain is associated with physical inactivity in older people. While walking is an acceptable form of physical activity, the effectiveness of walking interventions in this population has yet to be established.

**Objectives:**

To assess the acceptability and feasibility of conducting a randomised controlled trial (RCT) to test the effectiveness of a healthcare assistant‐led walking intervention for older people with persistent MSK pain (iPOPP) in primary care.

**Methods:**

A mixed method, three arm pilot RCT was conducted in four general practices and recruited patients aged ≥65 years with persistent MSK pain. Participants were randomised in a 1:1:1 ratio to: (i) usual care, (ii) usual care plus a pedometer intervention, or (iii) usual care plus the iPOPP walking intervention. Descriptive statistics were used in an exploratory analysis of the quantitative data. Qualitative data were analysed using thematic analysis. A triangulation protocol was used to integrate the analyses from the mixed methods.

**Results:**

All pre‐specified success criteria were achieved in terms of feasibility (recruitment, follow‐up and iPOPP intervention adherence) and acceptability. Triangulation of the data identified the need, in the future, to make the iPOPP training (for intervention deliverers) more patient‐centred to better support already active patients and the use of individualised goal setting and improve accelerometry data collection processes to increase the amount of valid data.

**Conclusions:**

This pilot trial suggests that the iPOPP intervention and a future full‐scale RCT are both acceptable and feasible. The use of a triangulation protocol enabled more robust conclusions about acceptability and feasibility to be drawn.

## 1 INTRODUCTION

Older adults generally have low levels of physical activity, which contributes to increasing morbidity related to long‐term conditions (LTCs) and loss of physical function (LaRoche et al., 2018; McPhee et al., 2016). Previous research examining the relationship between pain and physical activity highlighted that older adults with musculoskeletal (MSK) pain were significantly less active than those without MSK pain (Smith et al., 2021; Stubbs et al., 2013) and that pain is an important predictor of physical inactivity (Plooij et al., 2012).

Regular physical activity has been found to decrease pain and improve quality of life and function and reduce the risk of further LTCs in those with MSK pain (Ambrose & Golightly, 2015; Der Ananian et al., 2006). An overview of Cochrane reviews that examined physical activity for chronic pain in adults found some (but inconsistent) benefits for pain severity and physical function, although these were mostly of small‐to‐moderate effect, and, overall, the quality of the evidence was low (Geneen et al., 2017). Robustly designed randomised controlled trials (RCTs) of primary care‐based physical activity interventions that include objective measures of physical activity are required (O’Connor et al., 2015; Saragiotto et al., 2020).

Interventions that promote walking have the potential to be successful since walking is perceived to be popular, convenient, and inexpensive (Amireault et al., 2018; Matthews et al., 2016; Ogilvie, 2007; White et al., 2013). While previous walking interventions designed for people with persistent MSK pain have been shown to be associated with significant improvements in clinical outcomes, their long‐term effectiveness is uncertain (O’Connor et al., 2015). Many of these interventions may also be unsuitable for delivery in UK primary care due to complexity and the number and duration of contacts needed.

A brief walking intervention offered in primary care and designed for older people aged 60–75 years demonstrated increased step‐counts in the short and long‐term (Harris et al., 2015, 2018), and a subsequent RCT highlighted that hours of support from a healthcare professional (i.e. practice nurse) might not be required to achieve increases in walking in inactive adults between 45 and 75 years of age (Harris et al., 2017, 2018).

Whether these findings are transferable to older people with persistent MSK pain is unclear, therefore this study aimed to examine the acceptability and feasibility of future full‐scale RCT testing usual care versus usual care plus a pedometer and usual care plus the iPOPP intervention, which is a brief and simple walking intervention supported by healthcare assistants (HCAs) working in general practice (who work closely with general practitioners and general practice nurses in UK primary care) in older adults with persistent MSK pain in primary care.

### 1.1 Objectives

1. Determine the feasibility of recruiting general practitioners to participate in a RCT
2. Determine the feasibility of recruiting, training and retaining HCAs to deliver the iPOPP intervention
3. Examine the rate of recruitment to the pilot trial
4. Examine the response to the 12‐week follow‐up questionnaire
5. Examine adherence to the iPOPP intervention
6. Assess the acceptability and fidelity of the iPOPP intervention
7. Determine the feasibility of collecting objective physical activity data, using accelerometers, as the potential primary outcome for a future full‐scale trial

### 1.2 Pre‐specified success criteria

To determine the feasibility of a future full‐scale trial, the following success criteria were set:

1. Participant recruitment rates of at least 70% of the target (*n* = 105/150)
2. Follow‐up rates at 12 weeks of at least 70% of those randomised
3. An acceptable intervention adherence rate (at least 50% of those receiving the HCA‐supported iPOPP intervention to attend both consultations)
4. Satisfactory intervention acceptability scores at follow‐up, whereby the score for participants randomised to the HCA‐supported iPOPP intervention should be at least 5/10

## 2 METHODS

This study is reported according to the CONSORT (Eldridge et al., 2016) and TIDieR checklists for pilot trials (Hoffmann et al., 2014) and the GRAMMs checklist for mixed method studies (O’Cathain et al., 2008) (see Supporting Information S1, S2 and S3). The methods are summarised here and described in full in the iPOPP pilot trial protocol (Healey et al., 2018).

### 2.1 Design and setting

A three parallel‐group, multi‐centre pilot RCT with mixed‐method data collection was conducted in four general practices across Cheshire and the West Midlands, UK. Quantitative data were collected to identify participant characteristics, describe levels of acceptability and determine the extent of intervention fidelity. Qualitative data were collected to investigate HCA experiences of delivering the iPOPP intervention and the participant experiences of receiving the iPOPP intervention.

### 2.2 Randomisation and allocation concealment

Participants were individually randomised in a 1:1:1 ratio to one of three intervention arms using random permuted blocks, stratified by general practice, with support from Keele Clinical Trials Unit (CTU). The study statistician was blinded to the intervention allocation.

### 2.3 Participants and recruitment

Medical records at the 4 general practices involved in the study were searched to identify adults aged 65 years and over who had consulted their general practice in the last 12 months for MSK pain in one or more of the index sites (foot, knee, hip, back, shoulder or neck). These patients were sent a screening survey to determine whether they had persistent pain (≥3 months) and a chronic pain grade score between 2 and 4 (Von Korff et al., 1992). Those who responded to this survey, met the eligibility criteria and agreed to further contact were posted an iPOPP trial pack, which included an invitation letter, consent form, and baseline questionnaire. Patients were then contacted by a research nurse by telephone to discuss the study, confirm eligibility and consent to participate. Patients were asked to return the completed consent form and baseline self‐reported questionnaire. Each participant was then sent a waist worn accelerometer (wGT3X‐BT monitor, Actigraph) in the post to wear for the 7‐day baseline data collection. Once the accelerometer was returned to the study team, participants were randomised.

### 2.4 Interventions

The intervention arms are summarised below, and full details can be found in the protocol paper (Healey et al., 2018):

*Usual care*: Participants were sent high‐quality written information in the form of the pain toolkit (http://www.paintoolkit.org) in the post by the study team and continued to be managed via usual care. The pain toolkit is a simple booklet that provides participants with tips and advice to support them in managing pain. Usual primary care management normally consists of a patient consulting their GP or practice nurse for their pain and may include advice and education, the prescription of medication and referrals to other appropriate services, such as physiotherapy, podiatry or occupational therapy. No restrictions were placed on other healthcare. It could also mean no further intervention beyond their index consultation in primary care through which they were identified as potentially eligible for this study.

*Usual care plus pedometer*: Participants were mailed the pain toolkit, a pedometer, a pedometer user guide (based on the NICE guidance on promoting walking http://www.nice.org.uk/guidance/ph41) and a walking diary (NICE, 2012). No restrictions were placed on other healthcare.

**FIGURE 1:**
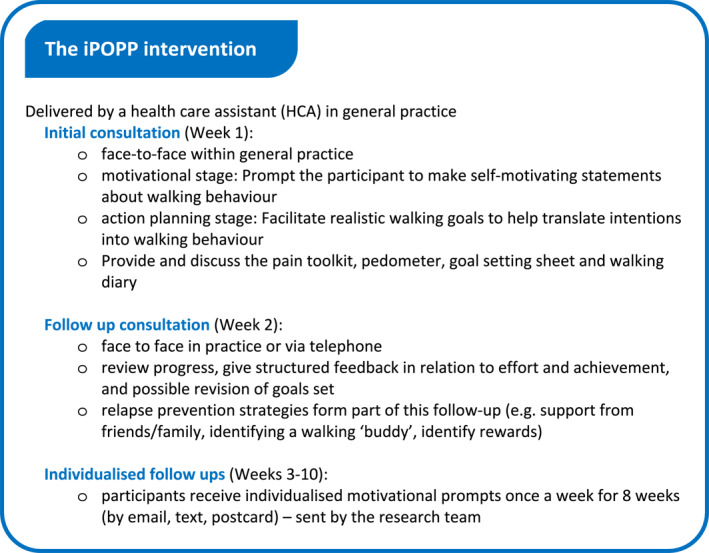
Summary of the iPOPP intervention.

*Usual care plus iPOPP*: The iPOPP intervention was developed in line with the Medical Research Council's framework for the development and evaluation of complex interventions (Craig et al., 2008), builds on previous research and is underpinned by explicit theory (Bandura, 1986). In summary, the development of the iPOPP intervention involved (i) a rapid review of the literature to identify effective interventions to build self‐efficacy to increase walking behaviour in primary care based populations; (ii) a stakeholder workshop (*n* = 10) (including clinicians, HCAs, policy makers, patients and public health professionals) to investigate the feasibility of implementing the proposed intervention in primary care and explore potential barriers and facilitators to delivery; and (iii) a Nominal Group Technique workshop (Manera et al., 2019) to refine and agree the intervention components with representatives from the target population (*n* = 11), focusing in particular on ‘what’ should be delivered and ‘how’ it should be delivered (Healey et al., 2018; Jinks et al., 2016). No restrictions were placed on other healthcare. The iPOPP intervention is summarised in Figure 1.

### 2.5 HCA training

A focus group with HCAs (*n* = 5) was conducted to help identify and explore factors that would influence the delivery of the iPOPP walking intervention by HCAs and identify what the trial training programme needed to include. In line with previous research, the development of the training programme consisted of four phases and was guided by the Theoretical Domains Framework (TDF) (Jinks et al., 2016; Michie et al., 2008; Porcheret et al., 2014). The training took place in a group setting over 2 days, 1 week apart, to allow the HCAs to absorb and reflect on what they had learnt, re‐read intervention materials and complete ‘homework’ before further review and training.

### 2.6 Sample size

As this was a pilot and feasibility trial, a formal sample size calculation was not appropriate. However, for pilot studies, it has been recommended that the dataset should comprise a minimum of 30 participants in each intervention arm (Shih et al., 2004). We anticipated that the combined total loss to follow‐up to the iPOPP intervention would be no more than 30% at the 12‐week follow‐up time‐point and therefore aimed to randomise 50 participants to each intervention (total baseline recruitment of 150).

### 2.7 Data collection and analysis

To enable us to address the objectives set and to determine whether we achieved the success criteria targets, data collection consisted of both qualitative and quantitative data, summarised below (see Healey et al. (2018) for more details).

*Self‐reported questionnaires*: All trial participants completed a self‐report questionnaire at baseline and 12‐weeks post‐randomisation. Baseline questionnaire data provided information on participant characteristics (e.g. gender, age, pain sites, co‐morbidity, employment status). The baseline and 12‐week follow‐up questionnaires included measures of physical functioning and mental health, pain intensity, pain location, physical activity, and self‐efficacy for exercise. The 12‐week follow‐up questionnaire also collected data on participants' experience of adverse events during the pilot trial, and acceptability of the interventions.

Case *Report Forms*: All HCAs were asked to complete a case report form (CRF) for each participant to record what happened during the iPOPP consultations. CRFs were audited by the research team during the delivery phase of the intervention to ensure that the iPOPP intervention was being delivered per protocol and to identify any further training requirements for HCAs; and at the end of the pilot trial to help assess the fidelity of the iPOPP intervention delivery.

*HCA pre‐ and post‐training questionnaires*: All HCAs that attended the training were also asked to complete a pre‐ and post‐training evaluation questionnaire. Both questionnaires focused on assessing knowledge and confidence to deliver the iPOPP intervention.

*Accelerometry*: This pilot trial was not powered to detect differences in walking between the intervention arms. However, we collected data on the feasibility of using waist‐worn accelerometers (wGT3X‐BT monitor, Actigraph, FL, USA) to objectively measure levels of physical activity via average daily step count as the potential primary outcome for a future full‐scale trial by examining the return rate and percentage with valid data.

*Audio‐recorded iPOPP consultations*: A sub‐sample of first and second HCA iPOPP consultations was digitally audio‐recorded with HCA and participant consent. Audio‐recordings were used to assess the fidelity of the iPOPP intervention delivery.

### 2.8 Analysis

Analysis of the quantitative data was conducted by a blinded statistician and was exploratory and descriptive to provide evidence of acceptability and feasibility. The research team scored the content of the audio‐recordings against a pre‐specified fidelity checklist (see Supporting Information S4). This allowed intervention fidelity to be explored by examining what happened during the consultations.

### 2.9 Qualitative data

*Semi‐structured interviews*: Interviews with pedometer and iPOPP arm participants explored the experiences and acceptability of the interventions. The views of the HCAs on the content and usefulness of the training and the acceptability and feasibility of the iPOPP intervention were also explored. Pedometer and iPOPP intervention participants were recruited for these interviews after the follow‐up data (accelerometry and follow‐up questionnaire) had been collected at 12 weeks. All HCAs were invited to participate in individual telephone or face‐to‐face interviews at the end of the intervention delivery period. All participants gave written informed consent prior to their interview. Topic guides were used to guide and stimulate dialogue in the interviews. All interviews were digitally recorded with consent and then professionally transcribed verbatim. Purposive sampling ensured a range of age, gender, pain scores and self‐reported baseline activity level.

### 2.10 Analysis

The interviews were analysed using a constant comparison approach (Glaser, 1965; Hallberg, 2006). A framework approach was also used to facilitate the interpretation of the data (Ritchie & Spencer, 1994). Themes were then mapped to behavioural theory by using the TDF (Michie et al., 2005).

### 2.11 Mixed method triangulation protocol

A mixed method triangulation protocol was employed to merge data analysis and identify the complementarity of findings. This enabled the research team to draw robust conclusions about acceptability and feasibility and any changes required to the iPOPP intervention or trial processes to inform a future full‐scale trial.

The triangulation protocol employed was based on previous research and current recommendations (Aschbrenner et al., 2022; O’Cathain et al., 2010; Tonkin‐Crine et al., 2016), and was implemented to integrate the results of this mixed methods pilot trial after all datasets had been individually analysed (see Supporting Information S5). Three forms of triangulation were implemented, including method triangulation as multiple forms of methods of data collection were used (e.g., interviews, questionnaires and CRFs); investigator triangulation as between two and five researchers analysed the data across the different stages of analysis; and data source triangulation as data were collected through multiple participant groups, including trial participants and HCAs (Carter et al., 2014). The relationship between the datasets was identified as one of four outcomes: agreement, partial agreement, dissonance or silence.

### 2.12 Patient and public involvement and engagement (PPIE)

The School of Medicine at Keele University has considerable experience of involving the public in research and uses the NIHR UK Standards for Public Involvement (NIHR INVOLVE, 2019). Patient and public involvement and engagement (PPIE) was embedded in all stages of the iPOPP pilot trial and supported by our dedicated PPIE team. In line with INVOLVE's recommendations, the pilot trial concept, design, processes, patient facing documents, interview guide and the iPOPP intervention were all co‐designed with PPIE, which involved older adults with lived experience of persistent MSK pain. A lay co‐applicant was involved in the development of the funding application for this pilot trial and both the Trial Steering Committee (TSC) and the Trial Management Group included two lay members which ensured PPIE contribution for the duration of the pilot trial. Further details can be found in the protocol paper (Healey et al., 2018).

## 3 RESULTS

### 3.1 Quantitative data

#### 3.1.1 Recruitment

Four general practices were invited to participate in the trial; all four agreed and were involved for the duration. From these general practices, a total of six HCAs were recruited and participated in training. Five of these HCAs went on to deliver the iPOPP intervention and complete both the pre‐ and post‐training questionnaires.

**FIGURE 2:**
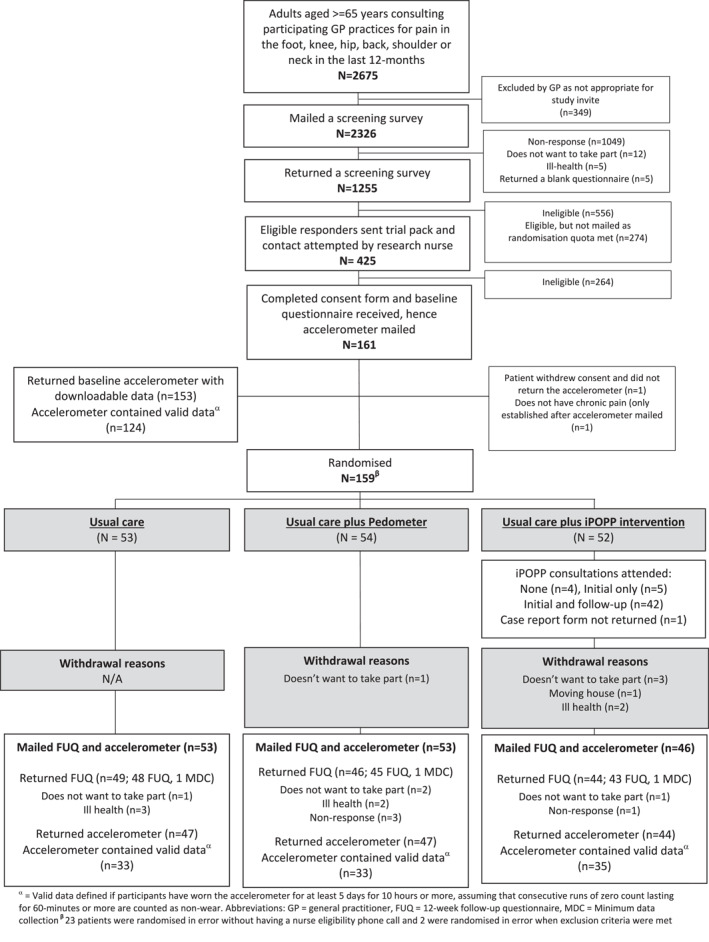
CONSORT flow diagram.

Of 2326 patients with MSK pain who were mailed a screening survey, 1255 (54%) responded and 699 (30%) were screened as eligible. After mailing trial information to 425 eligible participants, 161 (38%) agreed to participate, 159 returned a baseline questionnaire and were randomised (satisfying success criteria 1, see Figure 2). At this point recruitment ceased. Table 1 summarises the baseline self‐reported demographic and health characteristics and physical activity levels of the participants. Overall participant characteristics were similar across intervention arms.

**TABLE 1:** Self‐reported demographic and health characteristics from the screening survey and baseline questionnaire and baseline accelerometry data.

	Usual care *N* = 53	Usual care plus pedometer *N* = 54	Usual care plus iPOPP intervention *N* = 52	All randomised patients *N* = 159
Demographics				
Female gender	25 (47)	30 (56)	30 (58)	85 (54)
Age: Median (IQR)	73 (69, 79)	73 (67, 79)	71 (67, 75)	72 (68, 79)
Current employment status				
Retired from paid work	44 (92)	45 (94)	36 (80)	125 (89)
In paid work (full or part‐time, including self‐employed)	3 (6)	1 (2)	6 (13)	10 (7)
In full time education or training	0 (0)	0 (0)	0 (0)	0 (0)
Currently seeking employment	0 (0)	0 (0)	0 (0)	0 (0)
Voluntary work	0 (0)	0 (0)	1 (2)	1 (1)
Looking after home/family	0 (0)	1 (2)	2 (4)	3 (2)
Long term sick/disabled	1 (2)	0 (0)	0 (0)	1 (1)
None of the above	0 (0)	1 (2)	0 (0)	1 (1)
Index of multiple deprivation: Median (IQR) (Ministries of Housing, 2015)	23,431 (18,890, 29,984)	20,347 (16,071, 27,931)	25,439 (20,096, 28,586)	23,431 (17,301, 28,725)
Health				
Pain at any of the sites below lasting for ≥3‐months				
Foot	20 (38)	21 (39)	19 (37)	60 (38)
Hip	20 (38)	27 (50)	22 (42)	69 (43)
Knee	28 (53)	37 (69)	30 (58)	95 (60)
Back	34 (64)	28 (52)	27 (52)	89 (56)
Shoulder	23 (43)	24 (44)	18 (35)	65 (41)
Neck	18 (34)	14 (26)	16 (31)	48 (30)
Chronic pain grade (Von Korff, 1992)				
II (low disability‐high intensity)	25 (47)	26 (48)	26 (50)	77 (48)
III (high disability‐moderately limiting)	13 (25)	13 (24)	11 (21)	37 (23)
IV (high disability‐severely limiting)	15 (28)	15 (28)	15 (29)	45 (28)
Pain intensity, on average, in the last month (0–10): Mean (SD)	6.1 (2.0)	6.0 (1.9)	6.0 (2.3)	6.0 (2.0)
Past 4 weeks, pain lasting for 1 day or longer in any part of the body	47 (96)	49 (94)	46 (96)	142 (95)
Widespread pain^a^	11 (21)	7 (13)	15 (29)	33 (21)
EQ‐5D‐5L (−0.281 (extreme problems on all dimensions) to 1 (no problems on any dimension): Median (IQR) (Herdman et al., 2011)	0.81 (0.70, 0.84)	0.80 (0.67, 0.84)	0.79 (0.63, 0.89)	0.80 (0.67, 0.85)
Co‐morbidity				
Heart disease (including high blood pressure, angina, heart failure, stroke and heart attack)	25 (47)	32 (59)	34 (65)	91 (57)
Lung disease (including asthma, bronchitis and COPD)	9 (17)	15 (28)	5 (10)	29 (18)
Depression/Anxiety	9 (17)	6 (11)	9 (17)	24 (15)
Osteoporosis	6 (11)	9 (17)	11 (21)	26 (16)
Diabetes	6 (11)	6 (11)	6 (12)	18 (11)
Number of comorbidities: Median (IQR)	1.0 (0.0, 2.0)	1.0 (0.8, 2.0)	1.0 (1.0, 2.0)	1.0 (1.0, 2.0)
Physical activity				
Steps per day^d^: Mean (SD)	5079 (2446)	5223 (2857)	5352 (2851)	5221 (2716)
Percentage of valid time spent in^b^, ^d^: Median (IQR)				
Sedentary (0–99 CPM)	67 (62, 71)	64 (58, 69)	64 (55, 74)	65 (59, 71)
Light activity (100–1951 CPM)	31 (27, 36)	35 (30, 38)	34 (26, 42)	33 (28, 38)
Moderate activity (1952–5724 CPM)	1 (0, 5)	1 (0, 4)	1 (0, 4)	1 (0, 4)
Vigorous activity (5725–9498 CPM)	0 (0, 0)	0 (0, 0)	0 (0, 0)	0 (0, 0)
Very vigorous activity (>9499 CPM)	0 (0, 0)	0 (0, 0)	0 (0, 0)	0 (0, 0)
International physical activity questionnaire (IPAQ‐E): MET minutes per week: Median (IQR) (Hertig‐Wennlo, 2010)	1860 (800, 4905)	2079 (821, 4599)	3693 (1431, 7284)	2262 (1036, 5671)
IPAQ‐E single item: During the last 7 days, number of days spent walking for at least 10 min at a time				
0	1 (2)	2 (4)	3 (6)	6 (4)
1	3 (6)	2 (4)	1 (2)	6 (4)
2	2 (4)	4 (8)	6 (12)	12 (8)
3	3 (6)	6 (11)	4 (8)	13 (8)
4	1 (2)	2 (4)	3 (6)	6 (4)
5	8 (16)	4 (8)	4 (8)	16 (10)
6	2 (4)	4 (8)	3 (6)	9 (6)
7	30 (60)	29 (55)	28 (54)	87 (56)
On average, time spent walking per day in the last 7 days^c^: Median (IQR)	90 (35, 150)	90 (30, 120)	90 (60, 180)	90 (40, 150)
Self‐efficacy for exercise scale (Resnick & Jenkins, 2000) (0–10): Mean (SD)	6.0 (2.5)	5.9 (2.8)	5.7 (2.6)	5.9 (2.6)

*Note*: Figures are numbers and percentages unless otherwise stated. Valid time is calculated assuming that any consecutive runs of zero count lasting for 60‐min or more are counted as non‐wear. Accelerometry data processed using the default filter in the Actigraph software.

Abbreviations: COPD, chronic obstructive pulmonary disease; CPM, Counts per minute; IQR, Interquartile range; MET, metabolic equivalent; SD, Standard deviation.

^a^ Defined using the Manchester definition of widespread pain (Macfarlane et al., 1996).

^b^ Cut‐points from Freedson et al. (1998).

^c^ Time spent walking truncated to range between 10 and 180 min as per IPAQ scoring instructions.

^d^ Participants are only included if they have worn the monitor for at least 5 days for 10 h or more.

In terms of adverse events experienced during the trial, none were related to any of the trial interventions.

#### 3.1.2 Follow‐up

139 (87%) (usual care = 49 (92%), usual care plus pedometer = 46 (85%), usual care plus iPOPP = 44 (85%) participants returned the 12‐week follow‐up questionnaire and only 7 participants withdrew (satisfying success criteria 2) (See Figure 2). Those that did not respond at follow‐up were older, more likely to still be working and lived in more deprived areas. The greatest amount of missing data (18%) was found in the self‐report physical activity measure (IPAQ‐E) (Hurtig‐Wennlo et al., 2010).

152 participants were mailed an accelerometer at the follow‐up time‐point and 138 (91%) were returned (satisfying success criteria 2). 124 (78%) baseline accelerometers and 101 (67%) follow‐up accelerometers contained valid data.

#### 3.1.3 Intervention acceptability, adherence and fidelity

The CRF data demonstrated that of those randomised to the usual care plus iPOPP intervention arm, 47 (90%) attended the first HCA iPOPP consultation and 42 (81%) attended both consultations (satisfying success criteria 3). The median number of days between consultations was 7 days. The first and second iPOPP consultations took an average of 20 and 10 min, respectively. 36% of participants selected a face‐to‐face second consultation, while 64% preferred a second consultation via the telephone. Of those that reported a preferred mode for the motivational prompts, 58% preferred postcard, 15% preferred email, 27% preferred text (see Table 2).

**TABLE 2:** CRF data to assess the fidelity of the iPOPP intervention (51 CRFs returned).

	Initial consultation (*N* = 47)	Follow‐up consultation (*N* = 42)
Delivery of the iPOPP intervention		
Duration		
Consultation length: Mean (SD)	24 (9) (figures include one outlier with time = 60 min; all other consultation times range from 5 to 35 min; mode = 20)	13 (7) (range 5–40; mode = 10)
Preference of format for the 2nd consultation		
Face‐to‐face	16 (36)	N/A
Phone	28 (64)	N/A
Mode of weekly prompts		
Text message	11 (27)	N/A
E‐mail	6 (15)	N/A
Postcard	24 (58)	N/A
Content of the iPOPP intervention		
Prompt self‐motivating statements about walking	47 (100)	42 (100)
Address barriers/motivators to walking	47 (100)	42 (100)
Walking goals discussed	47 (100)	42 (100)
SMART walking goals set	46 (98)	39 (93)
Pedometer and user guide given	45 (96)	N/A
Walking diary given	43 (92)	N/A
Signposting	5 (local walking groups (*N* = 3); short walks to the shop (*N* = 1); already attends a walking group (*N* = 1))	No data given
Review progress	N/A	42 (100)
Review/amend goals	N/A	41 (98)
Relapse prevention strategies discussed	45 (96)	39 (93)

*Note*: Figures are numbers and percentages unless otherwise stated. The median number of days between the two consultations was 7 (range 6–24) based on 42 participants who attended both appointments.

Three HCAs recorded 18 iPOPP consultations (9 first and 9 second consultations). Fidelity checks (see Supporting Information S4) evidenced examples of good intervention fidelity by HCAs. The pedometer, user guide, walking diary and pain toolkit were all given, but there was a lack of explanation and discussion regarding the pain toolkit. Evidence of the benefits of walking for pain management was well communicated, motivators to walk and goals were discussed in all recorded consultations, with goals set in 7 of 9 first consultations.

### 3.2 Qualitative findings

**FIGURE 3:**
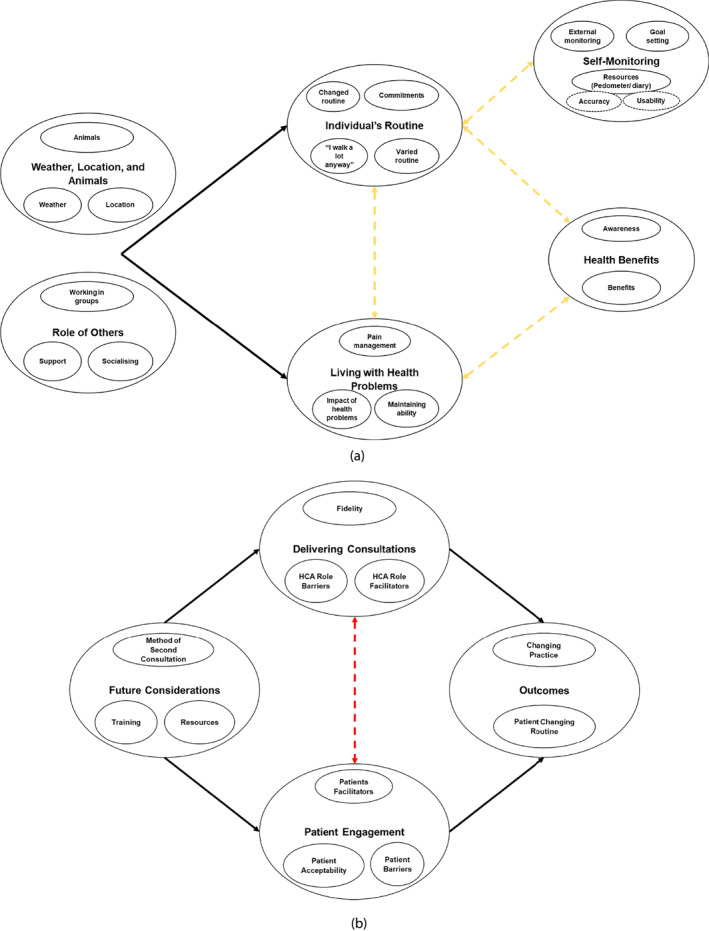
(a) Participant interviews thematic map. (b) HCA interviews thematic map.

Inductive analysis generated six themes from participant interviews and 4 themes from HCA interviews (see Figures 3a and 3b). In the participant dataset, there were shared themes across both the usual care plus a pedometer and usual care plus iPOPP intervention arms. Themes and subthemes with illustrative quotations are provided in Supporting Information S6. Facilitators and barriers, both from within and outside of the trial control, influenced engagement in increased walking activities. Important external facilitators include physical ability, flexible routines, nice weather, an appealing location, and having a purpose to engage in walking (i.e. walking a dog). Important external barriers included participants' feeling as though they lack the physical ability, busy routines (i.e. already feeling active enough, having support or work commitments), poor weather, and an unappealing location.

The main behavioural change technique discussed was goal setting (including reviewing goals). Both the pedometer and diary acted as vehicles to engage in goal setting. More usual care plus iPOPP intervention participants reported goal setting than usual care plus pedometer participants, suggesting the influence of attending HCA‐led consultations; though patients reported little involvement of the HCA in setting goals. Motivational prompts were not as commonly discussed by usual care plus iPOPP intervention participants, but they were reported as useful reminders, and in some cases, motivators to engage in walking activities.

Some participants raised concerns over the legitimacy of the pedometer, questioning whether the step count was accurate. Participants consistently reported not using and/or finding the pain toolkit useful; again, for the usual care plus iPOPP intervention participants, this may have been compounded by HCAs not fully explaining the toolkit during consultations as supported by HCA interview data.

HCAs' ability to deliver their role as an iPOPP intervention deliverer was influenced by facilitators (i.e. iPOPP training and resources) and barriers (i.e. patient activity level). Encouragingly, HCAs explained the usefulness of training, especially patient simulation, in feeling more knowledgeable, prepared and confident in delivering their role. Other iPOPP tools (i.e. laminate prompt card) acted as a prompt for HCAs within consultations. A key barrier, was the patient's perception of how active they were. Where patients were described as already being active, HCAs sometimes struggled; they would provide positive feedback on pre‐iPOPP activity levels but may not have utilised other BCTs within the iPOPP consultations. Additionally, HCAs reported second consultations being brief. This may highlight a lack of discussion and exploration on progress, goal adjustments, barriers and facilitators, and maintenance techniques. Importantly, for any future training considerations, HCAs illustrated examples of both good (exploring goal setting, exploring pain and exercise levels, and demonstrating the use of the pedometer) and poor (explanation of pain toolkit) fidelity when delivering iPOPP consultations.

#### 3.2.1 Findings from the triangulation protocol

In total, 65 findings were identified across the data collected (see Supporting Information S7). After comparing key finding statements against each other and merging duplicates and similar key finding statements, this number was reduced to 29 key findings. To ensure the completeness of the analysis, paired comparisons were performed, which resulted in 290 comparisons. Most of the comparisons (*n* = 241) resulted in silence, whereby only one dataset in the paired comparison contained data on a key finding. Of the remaining comparisons (*n* = 49), 22 (45%) demonstrated convergence between the datasets (agreement), 24 (49%) demonstrated data from one dataset complemented data from another (partial agreement), and only 3 (6%) demonstrated disagreement between datasets (dissonance) (see Supporting Information S8).

A combination of the quantitative and qualitative data showed that the HCA's felt able to carry out their role and had the appropriate knowledge to do so. HCA interview data illuminated elements which helped them to deliver the iPOPP consultations, including HCA intervention aids and training (which developed knowledge to share with patients; a better understanding of joint pain and that exercising with pain is safe and beneficial; instilling confidence and preparing them for delivering the consultation with participants). This was supported by the training questionnaire data where, for example, HCAs reported after training, feeling confident and competent to deliver the components of the iPOPP intervention, able to help patients identify facilitators and address barriers to maintaining/increasing walking, and capable of addressing resistance to increasing walking.

In contrast to the findings from the HCA training questionnaire and interview data, the audio‐recordings suggested that barriers to walking were not always discussed and exploration of maintenance strategies was lacking, especially with those patients who perceived themselves as already physically active. In agreement with the CRF data, all HCAs delivering the iPOPP intervention arranged a second consultation within 1 week, which was brief and took the form of a quick check‐up rather than a review of progress and focused mainly on use of the pedometer. While goals were re‐visited in most consultations, the HCAs did not always follow‐up to amend or revisit goals or explore the motivators/barriers to increasing physical activity.

A further example of agreement between datasets was in the interview data. Pedometer and iPOPP intervention arm participants reported the intervention components (e.g., pedometers, activity diary) as motivating them to walk throughout the trial. This was supported by HCAs who described consultations with participants and their own observations of diaries and the pedometer motivating iPOPP intervention arm participants.

Partial agreement often occurred when a dataset with data from all three treatment arms was compared with a dataset that did not contain data from all three treatment arms (e.g., qualitative interviews were drawn from iPOPP intervention and pedometer arm participants only). In terms of the acceptability of the interventions, the median (IQR) intervention acceptability scores (out of 10) from the 12‐week follow‐up questionnaire were, overall, higher for the usual care plus iPOPP participants (6.3 (4.3, 7.6) compared to usual care plus pedometer (5.0 (3.8, 7.8) and usual care only (5.1 (3.4, 7.1) participants (satisfying success criteria 4) (Borkovec & Nau, 1972). Data from the participant interviews complemented this and provided further details of acceptability as participants reported elements of the usual care plus iPOPP intervention which they found acceptable, such as the pedometer, the one‐to‐one approach with a HCA (i.e. not meeting in groups), and their GP practice as a location for their iPOPP consultation.

Across the paired comparisons, there were only a few instances of dissonance between datasets. When comparing interviews with trial participants against interviews with HCAs delivering the usual care plus iPOPP intervention, there were some differences in their views about goal setting. For example, usual care plus iPOPP participants reported setting their own goals instead of relying on HCAs to help them set goals, while HCAs reported examples of good intervention fidelity, including goal setting with participants in both consultations. Another example of dissonance highlighted the contrast between datasets as to whether participants increased their walking during the trial. In the interview data, participants who received usual care plus a pedometer or usual care plus the iPOPP intervention reported not increasing their walking. This contrasted with the HCA interview data and the physical activity data (self‐report and accelerometry), which suggested participants did increase their physical activity levels.

## 4 DISCUSSION

All of the pre‐specified success criteria were achieved in terms of general practice, HCA and participant recruitment, follow‐up (to 12 weeks), adherence to the iPOPP intervention and intervention fidelity and acceptability. The findings of this pilot trial also suggest a fully powered future full‐scale RCT comparing the clinical and cost‐effectiveness of usual care alone, usual care plus pedometer and usual care plus the iPOPP intervention would be acceptable and feasible with minor amendments to the research design and HCA training (detailed below).

### 4.1 Strengths

The use of a triangulation protocol within this pilot trial was novel and offered an additional process which enabled integration and more detailed interrogation of the mixed method data obtained (O’Cathain et al., 2010). This enabled a more complete analysis and deeper understanding of the findings, which went beyond those available from the analysis of the different datasets individually. For example, illustrating that the perceived lack of guidance from the HCAs by some iPOPP intervention participants (especially those already active) may be due to the uncertainty experienced by the HCAs around how to engage such individuals within the iPOPP consultations.

It is important to acknowledge that through the paired comparisons, the amount of evidence for each key finding varied, and therefore, so did the strength of the claim drawn about the key findings. For example, the analysis highlighted agreement that there were examples of both good and poor intervention fidelity within the data. However, the paired comparisons explicated richer and more frequent examples of good fidelity compared to poor fidelity across the datasets. Therefore, the triangulation protocol provided greater weight for different key findings, for example, providing confidence that overall the intervention was delivered well according to the intended protocol.

Our stakeholder and PPIE group that co‐designed the iPOPP intervention highlighted the importance of choice when it came to how participants received their consultations (i.e. face to face or by phone) and their motivational prompts (i.e. postcard, text, email). The results of this pilot trial reiterated the importance of this choice as participants selected the range of options we provided. This finding is supported by Loew et al. (2017) who reported that older people with knee osteoarthritis can increase adherence to walking programmes when supported according to their preference. It was interesting that 64% of those randomised to the iPOPP intervention preferred to receive their second consultation with the HCA over the phone. This is particularly helpful to know in the context of changes to health care delivery following the COVID‐19 pandemic, whereby telehealth utilisation has increased dramatically (Patel et al., 2021) and its use has persisted in some settings that seem particularly amenable to remote care, such as primary care (Mehrotra et al., 2021).

### 4.2 Limitations

The research team had envisaged that the triangulation protocol would present and analyse the data across all datasets together for each of the key findings. This would have allowed for a clearer output for each key finding where the overall relationship could be stated (e.g. agreement). However, as the team worked through the steps of the triangulation protocol, it was evident that this was not possible due to one or more of the datasets not providing data towards each of the key findings.

In terms of data collection, our follow‐up response rate for the self‐reported questionnaire at 12‐weeks was greater than expected, and overall the questionnaires were well completed and follow‐up was similar across interventions, all of which are encouraging for a future full‐scale RCT. However, we did identify that the self‐report physical activity measure (IPAQ‐E) demonstrated the highest levels of missing data. This is not of major concern as it was included as a back‐up measure of physical activity in case of issues collecting the accelerometry data (proposed future primary outcome). Overall, the return rate of the accelerometers was good at 12 weeks of follow‐up (91%); however, only 67% returned with valid data. The process through which accelerometry data is collected for a future full‐scale trial would need to be amended to maximise the amount of valid data obtained (see below).

### 4.3 Implications for research and practice

The results show that a future full‐scale trial appears to be acceptable and feasible and that general practices and HCAs could be encouraged to take part. The patient recruitment target was achieved rapidly within the recruitment period, suggesting that there is a real desire of older people with persistent pain to be involved in research and interventions based in primary care that are focused on providing support to be physically active. As stated above, follow‐up rates were very good for the self‐reported questionnaires at 12 weeks, although the accelerometry follow‐up at 12 weeks fell below our anticipated 70%. In a future full‐scale trial, we would adopt strategies to improve the response and the amount of valid data obtained. For example, a monetary incentive to return the accelerometers within a certain timeframe, amending the accelerometry user guide to increase the likelihood that they are worn correctly, relaxing the strict wear time criteria set (participants were only included in the analysis if they wore the monitor for at least 5 days, for 10 h or more each day) and only randomising those that provide valid data at baseline could all be considered.

Overall, the iPOPP intervention components were well delivered and the iPOPP HCA training was very well received with the HCAs feeling able to carry out their role delivering the iPOPP intervention and having the appropriate knowledge to do so. However, we have identified areas to optimise and refine the HCA training ahead of a future full‐scale RCT. For example, participants experienced differing external barriers and facilitators to increasing and/or maintaining walking activities. The HCA training therefore needs to consider these to further individualise consultations and embed walking activities into the participants' routines.

The study team also needs to consider ways of addressing the perceived lack of guidance from the HCAs by iPOPP participants, especially for those who believed themselves to be ‘active enough’. The data illustrate that behaviour change techniques, such as revisiting or amending goals set, may not have been implemented frequently. This highlights the need to develop motivational interviewing skills, through training, to support individualised goal setting and revision of goals.

Considerations also need to be given to how the usefulness of pain toolkit can be maximised. Participants sometimes reported not using the pain toolkit (though it was not always clear why it was not used); whilst some HCAs reported not explaining the toolkit in much depth.

## 5 CONCLUSION

This feasibility and pilot RCT demonstrated the acceptability of the iPOPP intervention, and the feasibility of trial processes, with all pre‐specified success criteria being met. A future full‐scale RCT is feasible and is needed to provide high‐quality evidence about the clinical and cost‐effectiveness of adding pedometers alone and the iPOPP intervention to usual primary care.

## AUTHOR CONTRIBUTIONS

Emma L. Healey, John McBeth, Elaine Nicholls, Carolyn A. Chew‐Graham, Stephen Dent, Nadine E. Foster, Elaine M. Hay, Liz Hartshorne and Clare Jinks were responsible for the concept and design of the study. Emma L. Healey, Daniel Herron, Elaine Nicholls, John McBeth, Liz Hartshorne and Clare Jinks supported the collection and analysis of the data. Emma L. Healey, Clare Jinks, Tamar Pincus, Carolyn A. Chew‐Graham developed and delivered the HCA training. Emma L. Healey prepared the manuscript with support and contributions from all co‐authors. All co‐authors approved the final manuscript.

## CONFLICT OF INTEREST STATEMENT

No conflicts of interest.

## TRIAL REGISTRATION

ISRCTN registry (ISRCTN: 10158028).

## ETHICS STATEMENT

All methods were carried out in accordance with relevant guidelines and regulations (e.g. Helsinki declaration). All participants provided written informed consent. Ethical approval was obtained from West Midlands ‐ Solihull Research Ethics Committee (REC reference: 15/WM/0329).

## Supporting information

Supporting Information S1

Supporting Information S2

Supporting Information S3

Supporting Information S4

Supporting Information S5

Supporting Information S6

Supporting Information S7

Supporting Information S8

## Data Availability

Data from this study are available on request from the first author.
