# Supplementary material for: The acceptability and feasibility of conducting a randomised controlled trial to test the effectiveness of a walking intervention for older people with persistent musculoskeletal pain in primary care: A mixed methods evaluation of the iPOPP pilot trial
Source: Musculoskeletal Care. 2023 Sep 9;21(4):1372–86. doi: 10.1002/msc.1815 (PMC10946998; doi:10.1002/msc.1815)
Supplement: Supplementary file 3 — Supporting Information S3 [file MSC-21-1372-s008.docx]

**Good Reporting of A Mixed Methods Study (GRAMMS) checklist: iPOPP Pilot trial**

| **Guideline Section:** | **Page** |
| --- | --- |
| Describe the justification for using a mixed methods approach to the research question | 13 |
| Describe the design in terms of the purpose, priority and sequence of methods | 13 |
| Describe each method in terms of sampling, data collection and analysis | 10-13 |
| Describe where integration has occurred, how it has occurred and who has participated in it | 13,18-20 |
| Describe any limitation of one method associated with the present of the other method | 22,23 |
| Describe any insights gained from mixing or integrating methods | 18-21 |

O'Cathain et al (2008)
