# Supplementary material for: The acceptability and feasibility of conducting a randomised controlled trial to test the effectiveness of a walking intervention for older people with persistent musculoskeletal pain in primary care: A mixed methods evaluation of the iPOPP pilot trial
Source: Musculoskeletal Care. 2023 Sep 9;21(4):1372–86. doi: 10.1002/msc.1815 (PMC10946998; doi:10.1002/msc.1815)
Supplement: Supplementary file 4 — Supporting Information S4 [file MSC-21-1372-s001.docx]

**
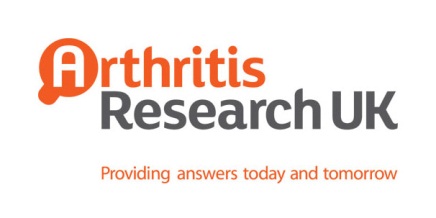

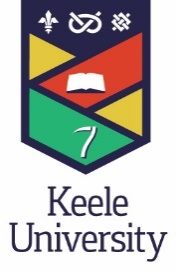
**

**Increasing physical activity in older people with chronic musculoskeletal pain: A brief and simple intervention to promote walking**

**Fidelity Checking Process – iPOPP Consultations**

The rationale for the fidelity process was to assess whether components of the consultation intended to be included, and focused on during training, were demonstrated by the HCA.

1. Fidelity checklist developed by Research Assistant
2. Fidelity checklist sent to members of the qualitative team to be reviewed and revised.
3. Appropriate amendments made.
4. Initial review of audio recordings checked for quality by Research Assistant
5. Qualitative team met to review a selection of the audio recordings and amend the fidelity checklist as required.
6. Qualitative team members collectively listened to a selection of the audio recordings and individually rated against fidelity checklist.
7. Joint discussion between members of qualitative team to ensure collective agreement of components met against the fidelity checklist.
8. Steps 5 – 7 repeated
9. Research Assistant reviewed the remaining audio recordings until all of the consultations had been rated against fidelity checklist.
10. Fidelity summary completed by Research Assistant.

iPOPP Qualitative

1^st^ Consultation Fidelity Summary

**Demonstration of the iPOPP intervention component**

| **HCA approach when conducting iPOPP consultation** | YES | Partially | NO | N/A |
| --- | --- | --- | --- | --- |
| Verbal explanation given for the iPOPP study (and check consent) |  | 8 | 1 |  |
| Although there was evidence of verbal explanation none of the HCAs checked consent with the participants. | | | | |
| The use of open questioning is evident throughout the consultation | 5 | 4 |  |  |
| Active listening is used throughout the consultation | 7 | 2 |  |  |
| Explanation of the evidence for the beneficial effects of walking with regards to pain | 6 | 2 | 1 |  |
| Barriers to walking explored | 3 | 1 | 4 | 1 |
| Motivators to walking explored and/or encouraged | 3 | 6 |  |  |
| 6 of these consultations were patient-led discussions around walking motivators. | | | | |
| Concerns about pain and/or increasing walking are acknowledged | 2 | 5 | 1 | 1 |
| Reassurance given as appropriate | 1 | 2 | 1 | 5 |
| Advice about who to contact if pain/joint problem worsen acutely | 6 | 1 | 2 |  |
| The participants were told to contact the GP or HCA no direct instructions or contact details were given. | | | | |
| Goals discussed | 8 | 1 |  |  |
| SMART walking goals set | 6 | 1 | 1 | 1 |
| SMART walking goals set with participant’s involvement | 5 | 1 | 2 | 1 |
| The goal setting was patient-led rather than a two-way patient centred consultation process. | | | | |
| Pain toolkit given | 9 |  |  |  |
| Pain toolkit explained |  | 2 | 7 |  |
| Pedometer and user guide given | 9 |  |  |  |
| Pedometer and user guide explained | 8 |  | 1 |  |
| Walking diary given | 9 |  |  |  |
| Walking diary explained | 8 |  | 1 |  |
| The focus during the consultation was on the explanation of the pedometer and diary. | | | | |
| Participant signposted to local opportunities– (e.g. walking groups etc.) | 1 | 5 | 3 |  |
| The consultations were not tailored to individual participant’s interests or goals. | | | | |
| Maintenance strategies explored (e.g. support from friends) | 1 | 3 | 5 |  |
| Maintenance/relapse strategies encouraged (e.g. support from friends) |  | 1 | 8 |  |
| If participant was already active the HCA found it difficult to advise on a maintenance strategy. | | | | |
| The participant’s understanding of what has been discussed and agreed is checked | 3 |  | 5 | 1 |
| 2^nd^ consultation arranged (in practice or telephone) | 9 |  |  |  |
| 2^nd^ consultation arranged within reasonable timeframe in line with protocol (approx. 1 week later) | 9 |  |  |  |

**Summary from 1^st^ iPOPP Consultation:**

- The majority of the consultations did not utilise the allocated time of 30 minutes. The average time the HCA spent delivering the 1^st^ consultation was 14 minutes and the range was 13minutes.
- During most of the consultations the HCA provided a brief verbal overview of the iPOPP study. However none of the HCA checked consent during the consultations.
- Whilst it was evident from all 9 of HCA interventions that the skill of active listening and the use of open questioning were used to some extent throughout, the consultations took the form more of a checklist assessment than that of a patient-centred consultation.
- 8 of the consultations demonstrated the HCAs knowledge regarding the research evidence of the benefits of walking.
- The HCAs focused on using the diary record as well as the pedometer and the majority of the consultations included some form of explanation and example of how to use them.
- Although the pain toolkit was given in all 9 of the consultations to the patient there was a lack of verbal explanation from the HCA to go with it.
- The setting of some form of walking or activity goal was apparent during 7 of the consultations this was patient-led rather that HCA guided. However there was a lack of evidence and exploration into maintenance strategies especially with those patients that already were involved in some form of physical activity.

2^nd^ iPOPP Consultation Fidelity Summary

**Demonstration of the iPOPP intervention component**

| **HCA approach when conducting iPOPP consultation** | YES | Partially | NO | N/A |
| --- | --- | --- | --- | --- |
| Walking progress reviewed | 9 |  |  |  |
| This was done in an informal way and just by a question of “how did you get on”? -rather than a structured review process. | | | | |
| The use of open questioning is evident throughout the consultation | 3 | 6 |  |  |
| Active listening is used throughout the consultation | 3 | 6 |  |  |
| Participant asked if they have used the diary (if applicable) | 4 |  | 5 |  |
| Participant asked if they have used the pedometer (if applicable) | 6 |  | 3 |  |
| Structured positive feedback given regarding effort and achievement | 7 | 1 | 1 |  |
| Walking goals revisited |  | 8 | 1 |  |
| This was done in a non-structured way and was not specific to the goal previously set. | | | | |
| Walking goals amended appropriately | 3 | 4 | 2 |  |
| Motivators to walking revisited |  | 8 | 1 |  |
| Motivators to walking encouraged | 4 | 2 | 3 |  |
| Barriers to walking revisited | 4 | 2 | 3 |  |
| Barriers to walking addressed |  | 5 | 4 |  |
| Participant-led rather than HCA initiated. | | | | |
| Any adverse effects discussed | 3 | 5 |  | 1 |
| Any adverse effects dealt with appropriately | 2 | 3 | 1 | 3 |
| Signposting – (e.g. walking groups etc.) | 1 |  | 7 | 1 |
| The consultations did not demonstrate individual participants activity interests. | | | | |
| Maintenance/relapse strategies discussed (e.g. support from friends) | 1 | 6 | 2 |  |
| Patient reminded to contact the iPOPP team if they have any issues or further advice is required regarding the study. Or to contact the GP practice if they have any clinical concerns/issues | 1 | 3 | 5 |  |
| Method of prompts agreed | 9 |  |  |  |
| Contact details were not confirmed for chosen method of prompt. | | | | |

**Summary from 2nd iPOPP Consultation:**

- The 2^nd^ iPOPP consultations were very short in time, the average time spent in the follow-up consultation was 6.5 minutes and the range was 8 minutes.
- The 2^nd^ consultation took the form of a quick check-up rather than a review of progress.
- The focus was on the use of the pedometer and although the patient goals were in most of the consultations re-visited, the HCA did not always follow-up from this with amending the goals or exploring the motivators to increasing physical activity.
- 8 of the 2^nd^ iPOPP consultations provided some form of exploration or discussion around the patients’ experience of adverse effects.
