# Supplementary material for: The acceptability and feasibility of conducting a randomised controlled trial to test the effectiveness of a walking intervention for older people with persistent musculoskeletal pain in primary care: A mixed methods evaluation of the iPOPP pilot trial
Source: Musculoskeletal Care. 2023 Sep 9;21(4):1372–86. doi: 10.1002/msc.1815 (PMC10946998; doi:10.1002/msc.1815)
Supplement: Supplementary file 5 — Supporting Information S5 [file MSC-21-1372-s006.docx]

Step 1.2. Develop key findings into finding statements

Step 1.3. Discuss and agree upon key finding statements by consensus

Step 1.1. Identify key findings for each dataset

**Step 1. Develop finding statements for each dataset**

**Step 2. Develop a final list of key finding statements for a convergence coding matrix**

Step 2.2. Collate and discuss outcomes of the comparison process until there is
 agreement on a final list of key finding statements

Step 2.1. Independently compare each key finding statement with all other statements
 across all five datasets

**Step 3. Identify relationship between datasets**

Step 3.1. Independently use paired comparisons to compare each dataset against each key
 finding

Step 3.2. Identify the relationship for each paired comparisons

Step 3.3. Relationships between the paired comparisons discussed and agreed upon by
 consensus

**Appendix 1.** Steps of the triangulation protocol
