# Supplementary material for: The acceptability and feasibility of conducting a randomised controlled trial to test the effectiveness of a walking intervention for older people with persistent musculoskeletal pain in primary care: A mixed methods evaluation of the iPOPP pilot trial
Source: Musculoskeletal Care. 2023 Sep 9;21(4):1372–86. doi: 10.1002/msc.1815 (PMC10946998; doi:10.1002/msc.1815)
Supplement: Supplementary file 6 — Supporting Information S6 [file MSC-21-1372-s007.docx]

| **Theme** | **Theme Memo** | **Sub-Theme** | **Example Quotes** | | **Sub-Theme Memo** | | |
| --- | --- | --- | --- | --- | --- | --- | --- |
| **Life with Health Problems** | All participants spoke about their life with a health problem; this usually related to pain, however, many participants had co-morbid conditions which impacted upon their health. Most participants were impacted upon by their health condition(s), usually through loss of mobility. This impact could also impede the participant’s engagement with iPOPP/ walking activities.  Participants described managing their pain through various techniques, such as medication, exercise, rest, and a combination of all. Important here, was that their understanding of their own body and how it reacted. They understood how to better manage the pain.  For participants, life with health problems meant trying to maintain the things they enjoy. Peoples’ abilities to do this varied on the severity of their health problem; however, they were determined to limit the impact it had on their ability to engage in activities, such as walking or golf. This motivation was underpinned by an understanding that being active was necessary to help manage/ control their pain; whilst for others, being active was a ‘mind set’- they had always been active and found it challenging to ‘sit in’, and being inactive, for example due to their pain, was not an option. Participants were determined not let their health problem control their lives. Pain could also impact upon the control and independence people had once previously enjoyed. Participants were resistant to this. | **Impact of Health Problems** | Yeah, [yeah] both of them [yeah]. It was only one at first and now it’s gone to both and it’s really difficult getting dressed and doing lots of things [right, okay]. So that’s my main problem; my upper body**(INT PX3)**  Yes, well I had two knees. I’m absolutely ar-, - er, riddled with arthritis. I’ve got it in my feet, [yeah] in my toes er, and in my hands [yeah]. So... [yeah] anyway... **(INT PX5)**  Yes, yes and I had to give up golf. I played golf [yeah] and er, I just couldn’t do it anymore and er, so yeah, it’s, it’s, it’s been okay but it’s slowly got worse [yeah, yeah] erm, so – but I would say ten years **(INT PX5)**  But, for some reason, it will suddenly give up on me [yeah]. You know, as though somebody’s cut my knee off, you know, and really limp. And then the next day it can be perfectly alright again **(INT PX8)**  The arthritis will, so what it does - one thing it does stop me doing is going for long walks . I’d like to walk more [yeah]. You know, whereas, I’m only walking in short bursts [yeah], 100 yards, you know, getting on a tractor or whatever [yeah, yeah]. I couldn’t - I couldn’t walk five miles **(INT PX8)**  Yeah [yeah] and, er, the pneumonia set me back [yeah], I had low bone pneumonia **(INT PX10)**  Because some days, some days were worse than others. They still are, some days are – I get up out of bed in the morning and sometimes it’s a good half hour before I can get going, get moving, you know, because of my hip and…. I still have the pain, you know **(PED PX1)**  My angina is my problem. Solely that is my problem. I mean my legs were so strong, I used to have an act where I could hang from a girder, which I have done, by my feet. I could hang by my feet and then my legs were strong enough to allow me to get back again and - but now they don’t **(PED PX2)**  The angina has absolutely ruined me bloomin’ life in many respects.  We’ve now set a gardener on. Fancy having to set a gardener on. Why can’t I do it? Hoovering is a problem. Using my sweeping brush, I don’t know why that is. I can pick a bag of cement up and dump it somewhere, but other problems give me **(PED PX2)**  It comes in bouts. So, I can have – I’m never completely pain free, so I wouldn’t get out of bed, for example, like most ordinary people would because it hurts to get out and to just sit up. So, I’m never really pain free. But it’s only on rare occasions that I’m in a lot of pain, I think I could honestly say **(PED PX4)**    Erm… No not really, because some days I find it quite difficult to walk, and other days you know I could walk miles **(INT PX6)** | | It was common for participants to have health problems which developed and were present prior and during the intervention. Most participants focused on their pain, which they attributed to arthritis, but many other physical health problems were discussed. Some participants described how the health problems they experienced were barriers to engagement in walking activities and/or the intervention. For instance, long term pain prevented people from walking; pneumonia, which developed during the intervention, or an unexpected admission to the hospital, prevented them from engaging in the iPOPP intervention. Pain in particular could be unpredictable, which sometimes made it difficult to manage. Prevalent across participant experiences of health was *impact*. Health problems impacted upon what the individual could do. Most participants spoke about loss of ability; something they could do prior their health problem, which they struggled to do now. This ranged from daily tasks, such as holding a cup, and putting clothes on, to being able to walk. | | |
|  |  | **Managing Pain** | Er, no, I don’t actually. Erm, I don’t like taking tablets. But, er, when it does get bad obviously you need to take something **(INT PX1)**  Er, walking, like I say, the swimming has helped, er, but, erm, I do know that if, if I, if I’m not moving about then, er, I, I do seem to seize up **(INT PX1)**  Yeah, I take a painkiller and just walk through it. I never take more than two a day because I don’t like painkillers. But I do need those two to **(INT PX2)**  Very rarely [yeah]. Very rarely. I’ve been given codeine and things like that, but I - I only take it if the pain is too bad and if I’m going to take it I’ll only take it at night before I go to bed so I can sleep [yeah, yeah ]. If I’ve got pain in the day time, I’ll work through it. **(INT PX7)**  I’ve no weight on my knees, I feel fantastic then [yeah]. So I try and get a good night’s sleep**(INT PX8)**  No. Well, you see, I used to – go back a few years I used to have lots of painkillers. I used to have these ibuprofen [yeah], paracetamols, then I’d have stomach balancers to [hmm, yeah] deal with that. I was taking all these pills. I still have to take blood pressure tablets cause you can’t – not those. And I just said to Wendy one day, ‘I’m not taking any more of these,’ [yeah] and I’ve been better without them. **(INT PX8)**  So it, it aggravates and when you go to sit down after a while of sitting down you’re not so bad but when you first sit down, that’s why laying down helps ‘cos you’re taking pressure off it **(INT PX9)**  But I think I’ve got to listen to me body and take it easy and then start getting back to it **(PED PX5)**  But I mean you know there's, taking tablets isn't the answer is it [no] I'll do it if I'm desperate but I have to be desperate you know **(PED PX7)** | | Participants spoke about how they managed their pain. A variety of strategies were used, which were generally underlined by the individual’s understanding of their own pain and body. They described methods which reduced their individual pain. For instance, participants’ understood what aggravated or eased their pain. For some, their pain was aggravated through activity, so they rested; for others sedentary behaviour aggravated their pain, so they kept active. However, for some, certain exercises aggravated pain, so they avoided these and took up others to keep active.  Medication was a common, and for many, necessary form of pain management. For some pain reducing medication was essential to get through daily activities; for others medication was not necessary, or they chose not to use it due to their dislike of medication. Many participants spoke about their dislike of using medication, and instead only used it when necessary, generally when their pain interfered with daily life (sleeping etc.) or became too severe. Some spoke about stopping using their medication and the benefits of this. Others spoke about the challenges of using multiple forms of medication.  There was an understanding by most participants, that being active benefited them- it helped them to manage their pain, whilst sedentary behaviour stiffened up their affected joints and decreased mobility; however, it was a balancing act which required a combination of the two- being active and resting when needed. | | |
|  |  | **Maintaining ability** | You see, as I say, it’s, I’ve had it a year or two now and I sort’a just walk through it, you know **(INT PX2)**  Yeah, I’m not going to give in [yeah]. This is going [yeah]. I’m not having it [laughter] **(INT PX3)**  But I try not to let it stop me [yeah stop you] because I don’t want to be inactive [yeah] I can’t be doing with that **(INT PX6)**  Yeah the only the thing that worries me is if I can’t walk. If I can’t get out and about then you know I would pursue it because I can’t not… I can’t be inactive, I can’t not do things **(INT PX6)**  They’re not stopping me doing the everyday things, so you just carry on basically **(PED PX5)**  I really do because I think it’s very easy – I mean I had a very, very bleak time just over 12 months ago and it was very easy to think I can’t do this and do nothing. But for me that’s not really an option because a) I don’t want to do nothing and b) because it doesn’t do me physically any good to do nothing **(PED PX4)**  Yeah. But you can’t sit down and say that you can’t do this kind of – otherwise life wouldn’t be worth living. Not for me anyway. Not for me. I’m not a sedentary person **(PED PX4)**    Um well no really because you know I think the main thing about this condition is you’ve just got to keep moving, you’ve got to keep active [yeah] you know you’ve got to motivate yourself and that's the thing that I think a lot of people can't do or don't want to do and you know you’ve just got to keep going you know because life's very precious and you don't want to finish it sitting in a chair all day [no, no] you’ve got to keep doing something. **(PED PX8)**  It depends on your mind set [it does – it does you’re right], I’m not a sitter abouter anyway you know I never have been you know I’ve always been a get up and do something bloke you know **(PED PX10)**  I’d rather be dead than not be able to do - live my life as I want to do it [yeah, yeah]. I’m not ready for that yet **(INT PX8)**  The worst thing that could ever happen to me, would be not to be able to do it [yeah, yeah] I mean if I ended up in a wheelchair or something. Erm, I s’pose one thing I should do is to have my knee monitored but [yeah] it doesn’t go too far **(INT PX8)** | | Many participants lived with their pain, realising that it may never completely go but may subside. However, they were determined not to let it control their lives: stopping them from engaging in daily life and the things they enjoy. Participants, as much as possible, wanted to maintain being active, their control of what they could do and their independence. Many participants illustrated an understanding that being active was necessary to help manage/ control their pain; for others, being active was a ‘mind set’- they had always been active and found it challenging to ‘sit in’, and being inactive was not an option.  Being inactive was generally associated with negative comments, especially for those who have been active all their lives. They did not want to become sedentary.  For those that ‘being active’ was a part of who they were, it was important to maintain this, even through pain/ health problems.  Mind-set was important, as for those who had always been active, losing this and being ‘stuck in doors’ was not an option. | | |
| **Routine** | All participants had their own routine. For many, walking activities were embedded within their daily routine prior to iPOPP. The person’s routine was influenced by the flexibility and variability of their routine. Most participants were retired, and in comparison to those which were still employed, their routine varied from day to day, and was largely more flexible. Whether walking activities were engaged with sometimes depended upon what they had planned for that day.  The commitments already embedded within the individual’s routine influenced the up taking of increased walking activity. Some participants already had busy routines, which consisted of a job, or caring for a family member. Subsequently, they felt unable to be flexible in their routine, and unable to introduce new walking activities or increase their current walking activities.  The participant’s perception of how active their current routine was, influenced whether they increased their walking activities in their present and future routine. This was encapsulated by participants who spoke about already being active, and consequently, did not feel it necessary to increase walking activities through new or current activities.  When participants changed their routine, to increase walking activities, they included new activities or increased activities which were established in their routine. For new activities, this meant embedding it as part of their present or future daily routine. A purpose to introducing increased walking activity into a routine aided its inclusion and maintenance. For instance, walking to the shops to get food for a meal had purpose. | **Varying routine** | Yeah. I go there twice a week. We haven’t been this week yet because we’ve been so busy, so, er, we haven’t been able to go but, er, I’d imagine when she gets in tonight she’ll want to go **(INT PX1)**  Yes I did I did because sometimes I just do a small walk but there was days when I got nothing else to do or was working in here as an example I hadn't been down to the Dams for years, obviously because I [yeah] you know so one day I thought I'd go down to the Dams **(INT PX4)**  If you asked me any questions I'd know what I'd been doing then and some days I would just do that or gardening or we'd probably be going shopping or something like that so I didn't do anything but there were days when I'd think, I've got nothing else to do, I'll go a long walk and I'll walk around [07.33] and all that and sometimes I'd just walk to the back Lane **(INT PX4)**  Well actually I’m looking for another day to do something, um, but I don’t know what **(INT PX4)**  I go to the gym four or five times a week [right] in the morning for an hour each time **(INT PX7)**  Yeah. Yeah. Yeah. I’m actually retiring at the end of the month so I will be doing more [right]. I might take another allotment on **(INT PX7)**  I do try to get out every day, even if I only go to the village to buy a paper or pop into the butcher’s or I’ll go into the village once a week to get my hair done. **(PED PX3)**  I think I did but I think it went sort of up and down and up and down depending how I felt and what I was actually doing **(PED PX7)** | | All participants discussed their daily routine and the activities embedded within it prior the iPOPP intervention. This varied across the spectrum. At one end, activities were less vigorous and included only daily housework. At the end of the spectrum, activities were more vigorous.  A commonality of these experiences was flexibility. Many participants were now retired, and/or their children had moved out of the family home, so they implemented a flexible routine, which could vary day-to-day but also enabled them to implement walking into their routine. | | |
|  |  | **Commitments** | But when, when she said ‘oh, I only do it on a Thursday’ well that was no…no, you know, I can't be in three places on a Thursday. Just unfortunate, that **(INT PX2)**  I can't really. Well, we do work, see? We work, we clean houses **(INT PX9)**  No, not really, ‘cos we go out, we work Monday to Thursday now **(INT PX9)**  No, there isn’t anything else I could fit in [laughter]/ erm, but I don’t seem to have the time at the moment. **(INT PX3)**  Um I suppose I did really, I tried, I started off with just say five minutes and then I tried to up it but it was really so hard because I say I've got my mum, so obviously have to do her shopping um hospital visits, doctors visits all of that so I found it really hard to do that day by day **(PED PX7)**  Well it's time I need, it's just time, available time to go out and do it because you know we're together all the time, shopping, hairdressers…**(PED PX8)**  Well no and I was fully aware of that but I'm not able to do that now because of my husband's you know health that's changed dramatically so that we can't go for long walks any more **(PED PX8)**  it wasn’t easy to do the walks as much as I could you know because my husband is not well **(PED PX8)**  the lower spine, has been really severe in the last two years so this last 12 months I've been backwards and forwards between the Haywood doctors and checks and tests **(PED PX8)** | | One factor which influenced whether the person embedded increased activity within their daily routine was the commitments and responsibilities already underpinning their routine. This presented through two areas: caring for a family member, and their job. When people worked, they sometimes felt too busy to then include new or increased walking activities into their routine. Likewise, the commitment of supporting a family member meant that it was not always possible/ desirable to increase walking activity and spend time away from the individual. | | |
|  |  | **Active Routine: ‘I walk a lot anyway’** | I didn’t feel that I really [yeah] needed to [yeah] increase my walking because I was probably walking much more than people who had problems [yeah] with their lower limbs [yeah] more **(INT PX3)**  Erm, to be honest, it would have been difficult for me to increase them **(INT PX3)**  Erm, there wasn’t anything that I didn’t like [yeah]. I was disappointed that it wasn’t to do with my upper body [yeah] where I have the most of my problems **(INT PX3)**  I was already doing quite a lot. No, I don’t think I’ve increased it since taking part in the study. **(PED PX4)**  I walk a lot anyway so I didn’t try and walk any more than I normally do because I walk a lot. **(PED PX6)** | | The participant’s perception of how active their current routine was influenced whether they increased their walking activities through new or increased walking activities, in their present and future routine. This was encapsulated by participants who spoke about already being active, and consequently, did not feel it necessary to increase walking activities through new or current activities. | | |
|  |  | **Changing Routine** | Yeah, intention is at least twice a week. Take the dog out every day, er, for a walk, and probably go shopping about three, four times a week. Get, er, ‘cos I do the meals at night with my wife still working [Yeah]. So, er, I go and get, er, ingredients for the meals and, er, cook at night. And it’s just like a general routine. You just keep, just keep that up, you know. **(INT PX1)**  It's got you into the habit of walking though, even when he'd stopped he still walked/ …what I'm doing now is every morning I'll get up, have a shower and then go out, and I'll go down Windmill Hill, along Little Lane, along Lytton Road and up Connolly [right okay] now okay going down bank and all that **(INT PX4)**  When we come back from holiday I said to him you know, instead of sitting in at night, we could go for a walk round the block just for half an hour for a walk every night **(INT PX6)**  Because at the moment I’m doing all the driving ‘cause he can’t drive at the moment. So, I’m doing all the driving, so if I just want to go around the shop I’ll walk around the shop instead of driving, **(PED PX1)**  Actually we have an exercise bike, it was Eddie's exercise, a home exercise bike and um we both started using that [oh good] and you know I try to do at least 100 pedals in the morning and then in late afternoon **(PED PX8)**  Yes when I saw one day I wasn’t walking I um just didn't use my car and walked down to Tesco. **(PED PX9)** | | Those participants who indicated that they changed their routine, to increase walking activities, did this by including new activities or increasing activities established in their routine. For new activities, this meant embedding it as part of their present or future daily routine. A purpose to the introducing increased walking activity into a routine aided its inclusion and maintenance. For instance, walking to the shops to get food for a meal had purpose. | | |
| **Self-Monitoring** | Monitoring walking activity was important for many participants. It enabled them to identify their present step count, and respond to this with future actions- in many cases, this meant setting higher targets or behaviour change. The pedometer was a positive tool for engagement in, and increasing walking activities; however, the legitimacy of the pedometer was questioned, as some participants felt it did not accurately measure their step count, and struggled to use it. | **Goal Setting** | Well, I knew I had to try these steps, erm, but to, you know, to do 10,000 steps is impossible for me. **(INT PX2)**  No I think it does make you do a bit more, because I thought if you do 5000 one day you think oh I’ll try and do 6 tomorrow [yeah] So yeah I think it does motivate you. **(INT PX6)**  Yes. I found that I was thinking, ‘Ooh, you’ve only done 1,000 steps today, what about doing a bit more tomorrow?’ **(PED PX3)**  Yes. I actually found that was a good motivator because some days I walk a lot more steps than others. So, today for example, I’ve walked 12,000 steps. That’s just on a walk, that’s nothing to do with around the house, so I’ll probably have done 14,000 to 15,000 steps. But with that, some days – if it was a really dreadful day and I hadn’t walked a long, long way with everything I’d think, ‘I’ve only walked 7,000 steps today, I must do better tomorrow.’ **(PED PX4)**  I checked on the internet and thought well how many steps am I supposed to do a day and obviously there's various things with that and I tried to go for the lowest limit on what they said that you could do because I could see it obviously, that gives you a goal doesn't it? **(PED PX7)**  Just you know assumed that they were doing 1,000 steps, it was enough, you know I would like to have see you know what you call low activity, what you call high activity **(PED PX7)** | | Many participants described setting themselves targets. These were generally informed by two sources: the iPOPP resources and/ or to a less extent, external sources, such as the TV or magazines.  Targets were developed in reaction to what a participant achieved in the present. For instance, if one day they reached 4,000 steps, the next day they may try to reach 5,000 steps. Subsequently, such targets motivated people to engage in and increase the distance of walking activities. However, as participant **PED PX4** illustrated, targets may be contextual: dependent on the individual’s circumstances at a particular time.  Additionally, a few participants highlighted their informational needs- they wanted to know was constituted a low step count and what constituted a high step count. | | |
|  |  | **External Monitoring** | Er, no, I don’t think I did. No. She said she would contact me, I think, but she hasn’t. **(INT PX1)**  No. After, I think, three weeks I took the numbers down and just give them to the receptionist to give her ‘cos she hadn’t rung me. So, erm, and that was it really **(INT PX2)**  No. No. She did say, after the first session, there would be another iPOPP survey or a – the, erm, pedometer, but nothing else. She didn’t say there’d be another appointment with her **(INT PX7)**  I don’t think so because I haven’t decreased my activity. I possibly increased it. So I don’t think there would be any point in it. Cause I’m quite self-motivated. **(INT PX7)**  No, because I think for me, I don’t need motivating, I’m motivated to exercise/ It might’ve done but only in the initial stages. I think I’d soon have gone back to thinking that I do this anyway and so why am I trying to do anymore? I might’ve done to begin with, if somebody had said I had to do 15,000 steps a day, because…. **(PED PX4)**  Erm I think for anybody that’s motivated, I think people will do it regardless of a study like this. I think the greatest help is probably needed by the people who maybe have never exercised, who aren’t motivated and probably just telling them to exercise wouldn’t be sufficient. They need their hand holding or they need to go to a walking group every other day. Something where they’re with somebody else to give them a reason to go and to do it. Because I think if you, if you’ve always done it, you don’t need a reason because you enjoy it **(PED PX4)**  No, I think I’m quite happy setting me own things **(PED PX5)**  Being the sort of person that I am that was fine, I think it helps sometimes if you talk to other people and you can encourage other people and encourage yourself really so…**(PED PX7)** | | For participants in the intervention arm, it was common for follow up consultations not to take place. Subsequently, their engagement in walking activities/ or iPOPP was not monitored and advice not given. Though, when second consultations did take place, they provided positive feedback.  It was common for pedometer arm participants to not want external monitoring from a HCA, nurse or doctor through the iPOPP study. They felt happy setting their own things, and did not feel that they needed motivation from external sources. | | |
|  |  | **iPOPP resources** | Yeah. Yeah. The stepper did help ‘cos obviously you’re thinking ‘well, I could, you know, I’ll take the dog out and go round the block. I need another thousand steps’ **(INT PX1)**  Erm, well, I just looked at it and I thought some, some days I thought ‘Gosh, I don’t think I’ve sat down and look I haven’t done that many’ you know. It just, it just showed me how much you would have to do to reach, to reach a high number. **(INT PX2)**  es, yes. It was good to see because I’d thought to myself, ‘Mmm, I haven’t done quite as much as I thought I’d done’ or [yeah], or I’d done more than I thought I’d done/**(INT PX3)**  No I think it does make you do a bit more, because I thought if you do 5000 one day you think oh I’ll try and do 6 tomorrow [yeah] So yeah I think it does motivate you **(INT PX6)**  So, I found it was a great motivator for that, to try and average – to try and at least get to a decent number of steps that you did every day . Also, to find out actually how many steps you do walk just around the house for example **(PED PX4)**  I don't think you're aware of how much exercise you don't do and with having the step meter that was a big thing for me and really to record how much I do and then think well maybe I can do a bit more today and try and add to it so from that point of view it was you know **(PED PX7)**  Yeah it absolutely it's good because it surprises you, it makes you realise you know you’ve got to do more. **(PED PX8)**  Yes when I saw one day I wasn’t walking I um just didn't use my car and walked down to Tesco. **(PED PX9)**  No, it hasn’t made a scrap of difference to me and my habits **(PED PX2)** | | The iPOPP resources (pedometer and diary) were generally experienced positively. Both tools were used to monitor step counts and informed participants’ targets. Both tools helped to raise awareness of their activity level, and in many cases, encouraged participants to respond with action. For instance, **PED PX9** highlighted behaviour change in reaction to a low step count- they replaced a car journey with walking. However, not all participants found the iPOPP resources beneficial, with some explicitly stating that they did not utilise the tools and/or the tools not influencing their habits/ routine. | | |
|  |  | **Accuracy** | Erm, well, I just looked at it and I thought some, some days I thought ‘Gosh, I don’t think I’ve sat down and look I haven’t done that many’ you know. **(INT PX2)**  I mean some of my readings are low and yet I was busy [yeah], you know, gardening and doing [yeah] but I wasn’t walking... **(INT PX5)**  so but I mean I wasn’t aware of how accurate it is, if you read all this information now that says they're not accurate um and I would like to have known how many steps [04.21]. **(PED PX7)**  I tested all these up and down the garden because a lot of them you’ve got set haven’t you [yeah] and all this business and you know within, on 60 steps there was only one or two so I thought come on... Well I went exactly a mile from here down road to Morrison’s you know the petrol garage well it’s 0.9 actually in the car so I followed we go in the car so I walked down there and like and people will think what’s he doing and I’m looking at all these things and I’m thinking so many steps I’ll be alright and none of them are the same [no] and some of them there’s as much as 200 and 300 steps difference **(PED PX10)** | | The legitimacy of the pedometer was a concern for some participants, who questioned how accurately it measured their step count. This may raise concerns for participants, who may gradually lose confidence in the use of the pedometer. | | |
|  |  | **Usability** | They’re just the same but, er, I lost, well, I didn’t lose it; I, I lost it for one week and then it turned up, I’d got, left it in one of my pockets. Erm, but then, er it, it broke, it just wasn’t working **(INT PX1)**  Yes. It did drop off now and again, that one, but…yes, that was much easier to wear than the other. Yeah. **(INT PX2)**  you couldn’t be accurate with [yeah] that because it did come off [yeah] or slipped down, you know, or done something silly [yeah]. They’re, they’re not very secure **(INT PX3)**  No, the belt didn’t cause me any problems. It did wriggle up [yeah, yeah] from the waist up to here [yeah]. Erm, no but the little one did. It kept coming off **(PED PX3)**  **...**so I didn’t really know [yeah] and I actually lost one [yeah]. They did send me another one [yeah] because erm – so you didn’t – you couldn’t be accurate with [yeah] that because it did come off [yeah] or slipped down, you know, or done something silly [yeah]. They’re, they’re not very secure, [yeah] I don’t think. I think maybe the best thing to do would be to put them in your pocket **(PED PX3)**  Yeah, there were only a couple of times that I was tottering around and I thought, ‘Ooh, I haven’t put me….’ Yeah, it was only a couple of times I did that **(PED PX 1)**  I did use it, but it did stop working part way through **(PED PX 2)**  Half the time I forgot about it **(PED PX 6)**  Yeah it was fine um I can't remember if it was the first or the second one um conked out on me part way through the study and I did inform them but they said it was aright…**(PED PX 8)** | | Usability issues may have impacted upon accuracy. Participants mentioned pedometers breaking or falling off. One potential issue is participants giving up on the pedometer. | | |
| **Role of Others** | Other people played an important role in the participant’s lives; whether that was by being there for the individual or motivating them to engage in physical activity. HCPs were also seen as an important form of support. Most participants described experiences of HCPs- this commonly related to the support they received in relation to their health needs, such as to reduce the pain they were experiencing or to improve their mobility. HCAs were commonly discussed positively, however, the support they provided was minimal for most intervention arm participants. Underpinning this was the belief held by participants about already being active enough, or being unable introduce activity (too busy or too much pain).  The social aspect of walking and exercise may also be an important conducive factor. Many participants highlighted their joy with engaging with others when walking or exercising. Though a few participants preferred to an individualistic approach. However, though the social aspect was conducive, many participants highlighted being less likely to participate in the iPOPP study if they were required to work in a group. | **Socialising** | | Erm, but it’s not much fun going, walking on your own/ but I find the exercise classes more enjoyable because you’re with people, aren’t you? **(INT PX2)**  Yes. I think, erm, it would be more helpful to me to go for a walk than a pedometer or anything thing, you know. To go actually…[walking with people] **(INT PX2)**  Yes. And it’s also the people that you meet, the social aspect of it [yeah] which was always really good **(INT PX6)**  You know, there used to be like a little clique of us in the fields with the dog and that. So… And we’d go miles up through the woods, up through Keele Woods… [yeah] up around there…**(INT PX6)**  because, er, you know, that’s what I think sometimes with retirement as well, see, erm, when we had the shop you had conversation, you know, people come in about the weather about all, and when, you know, we , I mean, our daughter’s moving out next week; she’s lived with us for ten years but, erm, you, you just miss that conversation **(INT PX9)**  it’s quite nice to do something with somebody else, even walking the dog. It’s nice to meet somebody else with another dog; they can have fun and you can just walk and chat **PED PX4)**  Oh I love being out, I love talking to people, I can't sit in the house molly coddling myself all day. **(PED PX6)** | | The social aspect of walking was raised as an important element of walking activities. Many participants enjoyed being out and talking to people, and saw this as a motivating factor to engaging in activity. The social aspect of walking activities may have been important to participants many of which were retired or had no family/ little contact with family, to compensate for the little social contact they have. |  |
|  |  | **Support (Family and Friends)** | | Er, yes. Er, when my daughter was here I increased them tremendously ‘cos she, she had a stepper and she was trying to do 10,000 steps a day [Right, okay] so, er, some days I was doing up to 8,000 with her, er, but not so much now but I do go to the gym with my granddaughter **(INT PX1)**  Yes, [yeah] and I’m mostly with people who are very motivated as well **INT PX3)**  Yeah. Well sometimes.. A couple of times I’ve walked with my husband and we go to the pictures in the evening. Get down walk to the pictures and walk back [oh gosh yeah] and it only takes half a minute – er [tut] – half a minute, half an hour **(INT PX6)**  Oh yeah, we go for a walk now and again . Yeah. Yeah. Yeah. We go - we were going to start - at the end of the month, when I’ve retired, we’re going to start going to the gym together because she likes swimming and I love the gym, so we can have a couple of hours in - maybe even a couple of hours a day down the gym if needed/ Yeah. I was at work and I was doing – cause my wife had one as well, you know, a bit of competition. Who had the most steps, you know? **(INT PX7)**  So I mean, we work together so if the, if I’m rough my husband can help me, see? So, er, we do try and it’s helped us really, **(INT PX9)**  But fortunately, my daughter and my son only live a mile away. They both phone every day… ‘Mum, are you okay? Do you want anything bringing in?’ Erm my son usually brings me a paper and what have you. Yes, we’re a close family  Yes. But my daughter often says, ‘Look, I’m going to town on Saturday, you’re coming with me and we’ll just potter about and perhaps have lunch in Sainsbury’s or something and….’ Or she’ll say, ‘Oh, you’re making a Shepherd’s pie, I’ll come.’ Well, my daughter and granddaughter usually come on a Thursday for a meal **(PED PX3)**  Yeah my daughters insisted that I wear this and I only had it this week. **(PED PX9)**  it was about three and a half mile plus I walked there and did sort of extra mile and a bit and then we went to Trentham just before Christmas and walked, er, there was only three blokes turned up there but you know three’s better than two ain’t it so we walked round the lake and up to the monument and had a good, er, good talk and a good laugh **(PED PX10)** | | Support was an important element of participants’ experiences; though this social support was not necessarily explicitly linked to iPOPP or walking activities. Particularly important were family and friends. Family and friends provided a form of social contact for people, but could also encourage them to engage in walking, for instance, leaving the house for lunch or to visit their family member’s home. Family and friends were also seen as motivating, as they encouraged their family member to increase their step count, for instance, by taking them to the gym or by competing with them.  The HCA was viewed positively in terms of their approachability, and a few participants appreciated the feedback and (little) guidance they provided. However, most intervention arm participants discussed not receiving guidance on walking or setting targets- underpinning this was the person’s perception that they were already active enough prior the intervention. In this instance, it appears that the HCA struggled to provide guidance. |  |
|  |  | **(HCA)** | | I didn’t feel that I really [yeah] needed to [yeah] increase my walking because I was probably walking much more than people who had problems [yeah] with their lower limbs [yeah] more **(INT PX3)**  Erm, to be honest, it would have been difficult for me to increase them **(INT PX3)**  Not really because she did say, ‘Oh, I’m not sure that there’s anything more I can add to what you’re doing’. **(INT PX3)**  Um …not that I can remember but… ‘cause I’m quite active really um…[yeah] Not that I can remember. We talked about dogs and horses **(INT PX6)**  Yeah, that’s right, because she seemed to think I was being pretty good anyway ,but I’m still going to the gym when – well, I’ve been going to the gym for 30 years now/ No. She said I ought to decrease it a bit really **(INT PX7)**  when I went there she said, ‘Well you’re doing everything right anyway. There’s nothing - no point in this.’ [Right] So – and maybe I expected too much out of it. **(INT PX8)**  She said, ‘there’s nothing more you can do than you’re doing now.’…she hadn’t given me full information because she said, ‘If you go and see somebody else, that’s all they’ll tell you; what you’re already doing.’ **(INT PX8)**  Yeah she was telling me about walking and trying to increase your speed at walking and that sort of thing yes so yeah. **(INT PX4)** | |  |  |
|  |  | **(HCP)** | | Yeah if I have any real problems we've got very good doctors and I go and speak to them [yeah] you know they might, they know, they understand our situation and our home life so we're lucky that we've got good doctors. **(PED PX8)**  I couldn’t walk and then something moved and then I, I went last week, erm, she’s given me physio and then I’ve got to go next week but with the cast he said they usually last about 12 months so, er, in 12 months’ time contact them and go back, see ? **(INT PX9)**  he [Doctor]advised me to get some shoes with a high instep, which these….These are some that I’d got anyway and only used to use them on odd occasions. But since I’ve been wearing them the pain has really **(PED PX1)** | | HCP were also a key form of support. Though not necessarily explicitly linked into increasing walking activities, the support they provided may have better enabled the individual to engage with walking activities. Most of the participants described some form of support they had received from a HCP whether that be from physiotherapists to help strengthen their muscles and joints, doctors to understand their pain/condition and to help alleviate it, or operations through hospital staff. |  |
|  |  | **Working in Groups** | | I probably wouldn’t be, erm, er, interested in taking part **(INT PX1)**  Erm, the same really [mmm]; although, I think it is helpful. I agree with you. If... erm, if you’re – I suppose I might have got a bit stressed or impatient [yeah] if everyone wasn’t [yeah] – if I was running round and they were just going like this [yeah, yeah] but I think it is helpful to see other people [yeah], particularly if you’re not very active [yeah]. It would encourage you [yeah] and I suppose those of us who are more active can – could be helpful [yeah] to the others. Erm... **(INT PX3)**  Yeah I don’t mind being part of a group, ‘cause you meet different people. Different people [yeah] yeah … Or like one-to-one, I’m not really that bothered. [yeah] But I mean in a group would be fine, I wouldn’t object to being in a group [yeah]. **(INT PX6)**  Less. No, I’m not a social person. I think as a family, we are a close family, but we’re individual as well. After they’ve been here for a few hours I’ll say, ‘It’s time for you to go home, isn’t it?’ **(PED PX3)**  No I wouldn’t work in a, I'd rather do it on my own. **(PED PX6)**  I think it's partly my personality, I'm quite a driven person, if I want to do something and I set my mind on going to do it I would do it so I don't need people as far as that's concerned **(PED PX7)** | | Working with others, as part of future iPOPP interventions, had mixed reviews. Some participants were impartial to working in groups, and did not see it as influencing their decision- though they believed it introduced positive elements- such as encouraging others to be more active, and socialising with new people. However, for many people, the prospect of working in a group discouraged their participation. This may be informed by the participant’s perception of themselves- ‘not a social person’ ‘a loner’; however, concerns were raised about others not contributing, and not needing support from a group as they were already motivated. This may highlight the individualised approach needed for such an intervention, as though the social aspect of working in a group may be beneficial, for some, it was not appealing. |  |
| **Environment and Animals** | The environment and animals of the individual influenced whether they engaged in walking activities or exercise. The location, if found appealing, could induce walking. The weather, especially wet and slippery conditions, was viewed as a barrier which discouraged walking. Interestingly, having animals brought purpose to walking- those with animals engaged in walking. This purpose appeared to nullify the discouraging impact of weather. | **Location** | | Well I suppose I like the country really, I like going out **(INT PX4)**  The fresh air… [yeah] I like the fresh air [yeah]. I like being out and I like looking at the trees and things like that. Nature really. I just like being outside. **(INT PX6)**  It’s on your doorstep here. And because there are briar fields all around – I mean Cheshire’s just salt. There are these little metal workings where there’s, you know, briars in pipes underneath. And all the farmers rent off [34:49], who own it. But nobody really stops you. You can go for miles, you know, down these tracks and nobody really stops you **(PED PX5)**  Especially round the area that you know so well. Er, I mean, I, I’ll drive to Trentham and walk round there **(INT PX2)**  well, I’ve been away twice while I’ve been on the study. Erm, I went to the Scilly Isles; so we walked round all the islands. **(INT PX3)**  I did, yes, eventually, and…. The last bit, we were on holiday, weren’t we? The one that I had just for the week, half of it was done here and the other half was on holiday. So, I did quite a bit of walking a couple of days **(PED PX1)** | | An appealing location was conducive of walking. For instance, many participants described being out in country, or holiday locations as encouraging them to engage in walking. |  |
|  |  | **Weather** | | A hell of a difference. Yeah. Yeah, the cold weather, everything’s in pain, erm, but er, as, as the warm weather comes yeah, it’s brilliant **(INT PX1)**  I don't like getting wet, I mean sometimes I'll, during the winter there was sometimes when I'd go down the road to do a full loop [yeah] I remember one day, I got there and everything you know it started snowing and I pulled my hood up and I was covered in you know but not it was, I don't like the cold I must admit but um no **(INT PX4)**  No. The only thing what stops me doing it is the weather because if it’s rainy I can’t go up the allotment or in the garden, because I do a lot in the garden as well. So that’s the only - that’s my only downfall **(INT PX7)**  Well, at first, I thought with the weather, with the walking, you know, it was a bit difficult because er he hadn’t been well and so it was difficult to get out walking **(PED PX1)**  The only time it changes is if – and we don’t get a lot – is if there’s snow and it’s very icy underfoot. Because then – I don’t like driving now, in the bad weather, particularly here we don’t get gritted roads and things here. And to walk on it, because we don’t get gritted roads, sometimes it’s a solid sheet of ice. But it happens to rarely. But I think we can say that….**(PED PX4)**  Er, what stops me, yeah well I’m not going to go out at my time of life and, years ago I’d be out rain or sleet and snow [yeah]. The main thing that stops us now, and both of us and it’s only memorable really because we don’t get a lot of it is when it’s slippy [yeah] because, er, I think all of a sudden we’ve become like not doddery but, I’m like a bit frightened of falling [yeah] you know what I mean **PED PX10)** | | Weather could impact on people in two ways: it acted as a barrier as people did not want to go out in bad weather, and/or it was sometimes associated with increasing the pain the person experienced. Though, bad weather did not always deter people from walking. Having a purpose to go out in bad weather, such as walking their dog or tending livestock, cancelled out the impact of the bad weather |  |
|  |  | **Animals** | | when I walked the dog I just kept the steps up more than anything, just concentrating on how many steps you were doing **(INT PX1)**  Oh yeah… Because you have nothing really to go out for. You know, when I had him I had to go out in the morning, the afternoon and the evening [yeah] You know you had to come what may weather wise or anything **(INT PX6)**  Because um I know through having the dog if I was a bit fed up, I used to say to him, come on put you in the car, and we’d go out, we’d go out for an hour, come back, fine, no problems. It is the fresh air I think **(INT PX6)**  Yeah. Also, you see, if you’ve got a lot of cattle - I keep about 150 cattle, that if – they’re telling you - you can’t just not feed them, can you? **(INT PX8)**  Oh, I think the dog is a great erm…. I think, to be quite honest, if I didn’t have the dog I wouldn’t walk as much as I do **(PED PX4)**  You don’t know them, you’ve never seen them before, but people with dogs automatically say hello and talk to one another because that’s what they do. Whereas people that haven’t got dogs just walk on by. Because dogs always run up to you, don’t they? They have a sniff around or bark at you **(PED PX4)** | | Having animals created a purpose for an individual to engage in walking activities- the person had to go out, to ensure the wellbeing of their animal. This was explicated by a participant who had lost their dog, and described having nothing to go out for, and decreased their walking. Walking their dog may have also brought social benefits, as one participant described how it enabled them to interact with other people. |  |
| **Health Benefits** | The theme health benefits refers to the benefits which engaging in the iPOPP study and activity in general. A few participants discussed how the iPOPP study had raised awareness of the need to be active, and to maintain this. Other participants illustrated the health benefits of being active; engaging in the iPOPP study/ and or exercise in general had brought health benefits, such as having more energy or reduced pain. | **Awareness** | | Erm, well, I just looked at it and I thought some, some days I thought ‘Gosh, I don’t think I’ve sat down and look I haven’t done that many’ you know. It just, it just showed me how much you would have to do to reach, to reach a high number  **(INT PX2)**  Erm, I’m so glad I was in. Erm... well, I definitely think it’s a good idea [yeah] and erm, it has made me feel more aware and that, that I should definitely keep [yeah] being... **(INT PX3)**  Er it made me think about walking a bit more and doing things **(PED PX3)**  really hard as well but I mean I do do it so I'm aware that I do need to do the exercise and that's it more or less brought it home really but I've got to do it, use it or lose it they say don't they? **(PED PX7)**  It was it made me realise you know I've got to do something because as you're getting older you’ve got to keep moving **(PED PX8)** | | A few participants illustrated how engaging in the iPOPP study raised their awareness of the importance of being active and maintaining this. |  |
|  |  | **Improved health** | | Which it does. It does, actually. The more you move about the, the more it does help. It keeps things lubricated **(INT PX1)**  like I say, it seems, I seem to have more energy from it now, so obviously I can get a little bit more done **(INT PX1)**  I try just to go, even if I’m feeling a bit tired or whatever. I go and you feel great once you get there [exercise classes] **(INT PX2)**  Oh yeah, I feel a lot healthier in as much as I think this new allotment, so I’m doing a lot more work [yeah] and, erm, my pain level’s gone down **(INT PX7)**  I don’t really know. It did ease the pain a lot after a few days of you doing it and getting used to it. **(PED PX1)** | | Some participants discussed how engaging in the iPOPP study/ and or exercise in general had brought health benefits. This included increased energy levels and reduced pain. |  |
