# Supplementary material for: The acceptability and feasibility of conducting a randomised controlled trial to test the effectiveness of a walking intervention for older people with persistent musculoskeletal pain in primary care: A mixed methods evaluation of the iPOPP pilot trial
Source: Musculoskeletal Care. 2023 Sep 9;21(4):1372–86. doi: 10.1002/msc.1815 (PMC10946998; doi:10.1002/msc.1815)
Supplement: Supplementary file 7 — Supporting Information S7 [file MSC-21-1372-s002.docx]

| **Pedometer and Intervention Arm Participants Interviews** |  | **HCAs Delivering Consultation Interviews** |  | **Consultations (Recordings)** |  | **4. Quantitative data: Key Findings** |  | **5. Training Data** |
| --- | --- | --- | --- | --- | --- | --- | --- | --- |
| **1.1.** Pedometer and intervention arm participants were motivated to increase walking by the pedometer and dairy. Some intervention arm participants were motivated by prompts to maintain and engage in walking. |  | **2.1.** HCA’s reported elements of the iPOPP study which helped them to deliver the consultation (including HCA intervention aids [useful prompts] and training [which developed knowledge to share with patients; a better understanding of joint pain and that exercising with pain is safe and beneficial; instilling confidence and preparing them for delivering the consultation with patients]. |  | **3.1.** In the 1^st^ consultations, HCAs demonstrated examples of intervention fidelity (partial to good) when sharing knowledge (including, explanation of iPOPP study; benefits of walking on pain; the pedometer, user guide, and diary; who to contact if pain acutely worsen; and [at least partially] signposted to local opportunities). |  | **4.1.** Recruiting patients via a postal survey was feasible. |  | **5.1.** HCA’s felt they had reasonable knowledge of common musculoskeletal problems |
| **1.2.** Pedometer and intervention arm participants reported factors other than iPOPP tools which motivated them to engage, increase, and maintain walking (i.e. personality; having an animal/s; family and friends; an appealing location to walk; opportunity to socialise with others; better weather). |  | **2.2.** HCA’s experienced barriers and challenges when delivering the consultation to patients, (including, finding it difficult to encourage a person with pain to exercise, when they were already active, or pain in other areas than lower limbs; delivering consultations with patient participants they know, as compared to patient participants they do not know; [1] wary of providing advice to patients as they were not governed like HCPs; and patients lacking interest in participating). |  | **3.2.** The use of active listening and open questioning was evident throughout all 1^st^ consultations, including acknowledging and addressing participant concerns with pain and increased walking. |  | **4.2**. Good questionnaire follow-up rates were achieved. |  | **5.2.** Knowledge of chronic pain and pain medication is lacking |
| **1.3.** Pedometer and intervention arm participants reported using strategies and resources to manage their pain (i.e. rest; using medication as a last resort; and Healthcare Professional support [HCP]). |  | **2.3.** HCA’s reported positive outcomes of the iPOPP study, including from their own participation (changing their own practice and the usefulness of iPOPP resources [walking group leaflet] for own patients) and [1] that of the patient (positive impact on walking for some) |  | **3.3.** Most 1^st^ consultations did not use the allocated 30 minutes |  | **4.3**. Accelerometry return rates were good, however not all participants wore the device for long enough to meet criteria for valid time. |  | **5.3.** HCA’s felt confident and competent to deliver the components of the iPOPP intervention |
| **1.4.** Some pedometer and intervention arm participants reported positive outcomes from engaging in the iPOPP study (including, raising their awareness of the benefit of increasing their PA; feeling healthier and less pain; and increasing their walking). |  | **2.4.** HCAs reported that diaries and the pedometer motivated patient participants to maintain and increase their walking. |  | **3.4.** All HCAs arranged a second consultation, within one week, which were brief (average 6.5 minutes) and focused on the use of the pedometer. |  | **4.4.** Recruitment and follow-up rates were largely similar across the four GP practices, and, if differences did occur, they were not systematic i.e. no one practice could be identified as being particularly poor at recruitment and follow-up. |  | **5.4.** HCA’s were confident using the behaviour change techniques |
| **1.5.** Most pedometer and intervention arm participants received the iPOPP tools, including, the pedometer, diary, pain and toolkit (and for intervention arm participants, prompts, in which they were asked their delivery preference). |  | **2.5**. HCAs reported examples of good intervention fidelity (including, goal setting and encouraging patient participants to engage in walking). |  | **3.5.** In the 1^st^ consultation, HCAs demonstrated good fidelity when providing patient participants with iPOPP resources (pedometer etc.), discussing motivators, and setting smart goals (most with participant involvement). |  | **4.5.** Key characteristics of patients were associated with whether participants responded to the postal survey, gave consent to further contact, gave consent to take part in the trial, or were lost to follow-up. Gender and pain location are common determinants of sample differences. |  | **5.5.** HCA’s understood the benefits of walking for chronic pain and were confident discussing this with participants |
| **1.6.** Most intervention arm participants (especially those already active/busy) found the HCA approachable, and received positive feedback from HCAs, but reported little other HCA involvement (suggestions of activities, maintenance etc.) including not receiving second consultations (some reported being happy with one consultation) |  | **2.6.** HCAs understood the aim of iPOPP. |  | **3.6.** In the 1^st^ consultation, HCAs focused on using the diary record and pedometer, and the consultation took the form of more of a checklist assessment than that of a patient centred consultation. |  | **4.6.** The majority of participants received an initial and follow-up consultation. They preferred the second consultation to be over the phone and to receive weekly prompts via postcard. |  | **5.6.** HCA’s were confident with goals setting in this population |
| **1.7.** Pedometer and intervention arm participants reported factors which influenced their ability and perception of what they were physically capable of doing, and engaging in walking (including their health and their routine [for instance, having prior commitments prevented increased walking; whilst flexible routines allowed walking to be implemented]). |  | **2.7.** HCAs reported experiencing challenges with completing the CRF. |  | **3.7.** In the 1^st^ consultations, HCAs demonstrated examples of poor intervention fidelity (including not: checking consent; discussing barriers to walking; providing a good explanation of the pain toolkit; exploring and encouraging maintenance strategies [especially patients who perceived themselves as already physically active]; and checking participants of what had been discussed and agreed). |  | **4.7.** The HCA's reported good intervention fidelity. |  | **5.7.** HCA’s felt able to assess a patient’s needs and physical function |
| **1.8.** Pedometer and intervention arm participants felt that an individual approach to walking using an HCA was acceptable (i.e. not meeting in groups). |  | **2.8.** HCAs reported a preference for a face-to-face consultation for second consultations. |  | **3.8.** In most 2^nd^ consultations, HCAs demonstrated intervention fidelity (full and/ or partial) (including re-visiting patients’ goals and barriers; maintenance strategies; encouraging motivators to walking; agreeing method of prompts; and amending walking goals). |  | **4.8.** All three treatment arms were acceptable and credible to participants, however average scores for this domain were slightly higher for the iPOPP intervention. |  | **5.8.** HCA’s felt able to help patient’s identify facilitators and address barriers to maintaining/ increasing walking |
| **1.9.** Some pedometer and intervention arm participants were optimistic about maintaining their walking. |  |  |  | **3.9.** The use of active listening and open questioning was evident throughout all 2^nd^ consultations. |  | **4.9.** There was no evidence to suggest that the randomisation procedure had been unsuccessful (observed differences in patient characteristics by treatment arm could arise naturally due to small sample size). |  | **5.9.** I am aware of the Pain Toolkit booklet and feel able to respond to patient queries |
| **1.10.** Some pedometer and intervention arm participants discussed embedding increased walking into their routine (having already done so, or planning to do so in the future) |  |  |  | **3.10.** In the 2^nd^ consultations, HCAs demonstrated examples of poor intervention fidelity (including not: always asking if participants used the diary pedometer; and signposting participants to local services). |  | **4.10.** There are some differences in the characteristics of randomised participants by GP practice, however sample sizes are small for this to be fully interpreted and reliable. |  | **5.10.** HCA’s know when to ask the GP for advice about patients with chronic pain |
| **1.11.** Some pedometer and intervention arm participants reported not increasing their walking. For some participants across both arms, already being active meant they did not feel a need to increase their walking (more in pedometer arm). |  |  |  | **3.11.** 2^nd^ consultations took the form of a quick check-up, where walking progress was informally reviewed. |  | **4.11.** Key questions with a high percentage of missing data include: employment status, EQ-5D, pain in the last 4 weeks, IPAQ, self-efficacy score and treatment acceptability and credibility questions. |  | **5.11.** HCA’s feel able to understand a patient’s concerns about chronic pain |
| **1.12.** Pedometer and intervention arm participants reported varying knowledge of the iPOPP and study’s aims. |  |  |  | **3.12.** 2^nd^ consultations provided some form of exploration or discussion around the patients’ experiences of adverse effects. |  | **4.12.** There is a small signal in the data that levels of physical activity and self-efficacy scores, and a reduction in pain, are observed between baseline and follow-up in all treatment arms. |  | **5.12.** HCA’s feel capable of addressing resistance to increasing walking in patients |
| **1.13.** A few pedometer and intervention arm participants were uncertain about the walking intervention being relevant to them. |  |  |  | **3.13.** During 2^nd^ consultations, structured positive feedback was generally given regarding effort and achievement. |  | **4.13.** Serious adverse events were recorded by 20 participants, however only one was deemed to be possibly related to the intervention. |  | **5.13.** HCA’s feel able to communicate with patients both face-to-face and by telephone |
| **1.14.** Some pedometer and intervention arm participants reported difficulties with using the pedometer and accelerometer (forgetting to wear them, concerns about pedometer accuracy, and stopped working during the trial). |  |  |  |  |  |  |  |  |
| **1.15.** Pedometer and intervention arm participants reported not finding the pain toolkit useful. |  |  |  |  |  |  |  |  |
| **1.16.** Some pedometer and intervention arm participants reported elements of the iPOPP study as acceptable (the pedometer, and for intervention arm participants, their GP practice as a location for their iPOPP consultation). |  |  |  |  |  |  |  |  |
| **1.17.** Most pedometer arm participants reported being happy with monitoring their own engagement in the study. |  |  |  |  |  |  |  |  |
| **1.18.** Pedometer and intervention arm participants reported engaging in goal setting, which motivated them to try and increase their walking; however, not all intervention arm participants relied on HCAs, within the 1st consultation, to set goals |  |  |  |  |  |  |  |  |

**Appendix 3: Table of all the key finding statements across data sources.**
