# Supplementary material for: The acceptability and feasibility of conducting a randomised controlled trial to test the effectiveness of a walking intervention for older people with persistent musculoskeletal pain in primary care: A mixed methods evaluation of the iPOPP pilot trial
Source: Musculoskeletal Care. 2023 Sep 9;21(4):1372–86. doi: 10.1002/msc.1815 (PMC10946998; doi:10.1002/msc.1815)
Supplement: Supplementary file 8 — Supporting Information S8 [file MSC-21-1372-s005.docx]

| **Appendix 4.** The 10 pairwise comparisons between the datasets for each key finding and the number of comparisons for agreement, partial agreement, dissonance, silence, and not applicable. | | | | | |
| --- | --- | --- | --- | --- | --- |
|  | **Qualitative interviews with trial participants** | **Qualitative interviews with HCAs delivering iPOPP** | **Audio-recorded iPOPP consultations** | **Case report forms** | **HCA pre and post-training questionnaires** |
| **Qualitative interviews with trial participants** | x | 1  Agreement (3)  Partial Agreement (3)  Dissonance (2)  Silence (10)  Not applicable (11) | 2  Agreement (6)  Partial Agreement (1)  Dissonance (0)  Silence (12)  Not applicable (10) | 3  Agreement (3)  Partial Agreement (2)  Dissonance (1)  Silence (18)  Not applicable (5) | 4  Agreement (1)  Partial Agreement (4)  Dissonance (0)  Silence (12)  Not applicable (12) |
| **Qualitative interviews with HCAs delivering iPOPP** | 1 | x | 5  Agreement (3)  Partial Agreement (3)  Dissonance (0)  Silence (8)  Not applicable (15) | 6  Agreement (2)  Partial Agreement (2)  Dissonance (0)  Silence (16)  Not applicable (9) | 7  Agreement (3)  Partial Agreement (3)  Dissonance (0)  Silence (4)  Not applicable (19) |
| **Audio-recorded iPOPP consultations** | 2 | 5 | x | 8  Agreement (1)  Partial Agreement (0)  Dissonance (0)  Silence (20)  Not applicable (8) | 9  Agreement (0)  Partial Agreement (5)  Dissonance (0)  Silence (6)  Not applicable (18) |
| **Case report forms** | 3 | 6 | 8 | x | 10  Agreement (0)  Partial Agreement (1)  Dissonance (0)  Silence (16)  Not applicable (12) |
| **HCA pre and post-training questionnaires** | 4 | 7 | 9 | 10 | x |
